# Rheumatoid arthritis severity is mediated by crosstalk between synoviocytes and mature osteoclasts through a calcium and cytokine feedback loop

**DOI:** 10.1038/s12276-025-01401-8

**Published:** 2025-02-03

**Authors:** Eun Sun Lee, Hyeong Jae Kim, Dongun Lee, Jung Yun Kang, Dong Min Shin, Jeong Hee Hong

**Affiliations:** 1 Department of Physiology, College of Medicine, Gachon University, Lee Gil Ya Cancer and Diabetes Institute, Incheon, South Korea; 2 Department of Health Sciences and Technology, GAIHST, Lee Gil Ya Cancer and Diabetes Institute, Incheon, South Korea; 3 Department of Dental Hygiene, College of Software Digital Healthcare Convergence, Yonsei University, Wonju, South Korea; 4 Department of Oral Biology, Yonsei University College of Dentistry, Seoul, South Korea

**Keywords:** Ion channel signalling, Bone, Rheumatoid arthritis, Tumour-necrosis factors

## Abstract

Fibroblast-like synoviocytes (FLSs) and osteoclasts are central cells in the maintenance of joint homeostasis. Rheumatoid arthritis (RA) is a chronic inflammatory disease of joints that induces cytokine-activated FLSs and progressive bone erosion. Interactions between FLSs and other cells, such as T cells and B cells, have been recognized in the development of RA. Here we hypothesized that calcium released from bone by mature osteoclasts might activate FLSs, which are also affected by inflammatory cytokines in the inflamed synovium. Osteoclastogenesis occurs in the presence of cytokine-stimulated FLS medium, and calcium released from the bone disc activates FLS migration. We first investigated the calcium and cytokine feedback loop between FLSs and osteoclast maturation. Moreover, by addressing the role of the sodium-bicarbonate cotransporter NBCn1 in osteoclastogenesis, we found that the inhibition of NBCn1 attenuated the infinite calcium and cytokine feedback loop between FLSs and osteoclasts. In a collagen-induced arthritis mouse model, the inhibition of NBC reduced the RA pathological phenotype and bone resorption area in the femur. These results suggest that modulation of the crosstalk between FLSs and osteoclasts by inhibiting the calcium and cytokine feedback loop could be considered to develop pioneering strategies to combat RA severity and dysregulated bone homeostasis.

## Targeting feedback loop reduces joint damage in arthritis

Rheumatoid arthritis is a painful condition that damages joints and bones. Despite treatment advances, some patients do not respond well to current therapies. Researchers explored a new approach to address this issue. They focused on calcium and cytokines in RA. The study involved 16 patients and used mouse models to understand how these elements affect joint damage. The researchers found that Ca^2+^ from bones and inflammatory cytokines increase the activity of fibroblast-like synoviocytes, cells that contribute to joint damage. They discovered that inhibiting a protein called NBCn1, which helps cells move, could reduce this harmful activity. They used a drug called S0859 to block NBCn1 in RA-induced mice, which decreased bone damage and inflammation. The study suggests that targeting NBCn1 could be a new way to treat RA, especially for those who do not respond to existing treatments. This summary was initially drafted using artificial intelligence, then revised and fact-checked by the author.

## Introduction

Rheumatoid arthritis (RA) is a systemic inflammatory disease that causes pain, stiffness, inflammation and tissue damage, such as joint swelling and bone destruction. Structural changes in joints have been observed in patients with RA symptoms^1–3^. The pathological characteristics of RA include synovial inflammation, joint swelling, fibroblast-like synoviocyte (FLS) hyperplasia, pannus formation and bone destruction^4^. Physiologically, multiple aspects of RA mechanisms have been elucidated, and several clinical advancements have reduced disease symptoms. However, the complexity of RA and the precise mechanism involved have not been fully elucidated.

The inflamed synovium consists of various signaling components, including those involved in the immune response, metabolism, signal transduction, transport and apoptosis^5^. Bone damage in patients with RA is also related to disease severity. The synovium directly contacts bone, and bone-erosive lesions are observed in patients with RA^6,7^. Although clinical treatments for RA improve these signaling activities, combination treatments often attenuate disease severity; however, some individuals are nonresponsive to these therapies^2^. Notably, clinical trials have yielded unsatisfactory results, including adverse effects, with combination therapies. Based on the existing knowledge regarding combination treatment and no responsiveness, we determined whether a convergent target exists in the current mechanisms of RA to treat the bone and inflamed synovium.

Ca^2+^ is a global signaling messenger. Notably, Ca^2+^ signaling plays a pivotal role in bone remodeling. Bone is a major source of Ca^2+^, and cellular Ca^2+^ stores, such as those in the endoplasmic reticulum or nucleus, are sources of signaling. Premature osteoclasts are differentiated upon stimulation with bone remodeling factors, such as macrophage colony-stimulating factor (M-CSF) and receptor activator of nuclear factor-κB (NF-κB) ligand (RANKL), and induce a type of cellular fusion termed osteoclastogenesis^8–11^. Subsequently, multinucleated giant cells of osteoclasts are formed, and the bone is resorbed^11^. We hypothesized that Ca^2+^ released from the bone might activate FLSs, which are also affected by various inflammatory cytokines in the inflamed synovium. Activated FLSs cause hyperplasia, cartilage degradation and bone destruction. Thus, in this study, we designed the experimental conditions to demonstrate that free Ca^2+^ derived from bone and inflammatory cytokines mediate circulating feedback signaling through the cytokine loops between FLSs and osteoclasts to exacerbate bone damage in individuals with RA.

Previously, we reported that inflammatory cytokine-mediated FLS migration occurs via the involvement of the electroneutral type (1 sodium/1 bicarbonate) of the sodium‒bicarbonate cotransporter (NBCn)1 (ref. ^12^). Treatment with the NBC inhibitor S0859 inhibited cytokine-mediated FLS aggressiveness. In the present study, we investigated a potential strategy against cytokine feedback loops in RA by modulating bicarbonate transporters. We determined the role of NBCn1 in osteoclastogenesis and whether the inhibition of NBC reduces the infinite cytokine feedback loop between FLSs and osteoclasts. Moreover, we verified the inhibitory effect of S0859 on bone damage in a collagen-induced arthritis (CIA) mouse model. Overall, modulation of the crosstalk between FLSs and osteoclasts through NBCn1 could be considered a pioneering strategy to attenuate RA severity and dysregulated bone homeostasis.

## Materials and methods

### Patient enrollment and synovial fluid collection

A total of 16 patients were enrolled, including 8 patients with RA and 8 patients with knee osteoarthritis (OA), all of whom provided informed consent to participate in the study and had effusion in the knee joints. Patients with RA met the 2010 American College of Rheumatology/European League Against Rheumatism classification criteria^13^; however, those taking biological disease-modifying antirheumatic drugs (DMARDs) were excluded. Synovial fluids were collected from patients with RA or OA, as described in ref. ^12^, using procedures approved by the institutional review board of Gil Medical Center (GAIRB-2019-018). The synovial fluid was centrifuged at 1,500 rpm for 15 min and stored at −20 °C until use.

### Measurement of the Ca^2+^ concentration in human synovial fluids

Synovial fluids obtained from patients with OA or RA were used to measure the concentration of Ca^2+^ with a Ca^2+^ colorimetric assay kit (MAK022, Sigma). The Ca^2+^ concentration was measured according to the manufacturer’s instructions. Synovial fluid samples from patients were loaded into a 96-well plate and mixed with a chromogenic reagent. The Ca^2+^ colorimetric assay buffer was subsequently added to the mixture, and the final mixture was incubated for 10 min at room temperature in the dark. The absorbance of mixture in each sample was measured at 575 nm. The absorbance was calculated using the standard curve shown in Supplementary Fig. 1.

### Total RNA sequencing, KEGG pathway enrichment analysis and GO enrichment analysis

RNA extracted from RA-FLSs in the presence or absence of tumor necrosis factor-α (TNF-α; MTA00B; R&D Systems, 10 ng/ml) was analyzed via total RNA sequencing. Kyoto Encyclopedia of Genes and Genomes (KEGG) pathway enrichment and Gene Ontology (GO) enrichment analyses were conducted to evaluate the RNA expression patterns. The RNA samples were selected for KEGG and GO analyses. The top 12 biological processes in the KEGG analysis are indicated by bars and represent specific genes corresponding to the osteoclast differentiation process. Ion channel- and transporter-related GO terms are indicated by circles, and their statistical significance is indicated by the color of the *P* value.

### Primary FLS culture

Primary human FLSs, which were isolated from the synovial tissues of human patients with RA and OA, were purchased from Cell Applications (human RA-FLSs, 408RA05a; human OA-FLSs, 408OA05a). For the isolation of mouse FLS (Ms-FLS) from C57BL/6N mice, the skin surrounding the knee joints was excised from 43 euthanized C57BL/6N mice. Synovial tissue was then collected and rinsed with Dulbecco’s phosphate-buffered saline (DPBS). The tissue was subsequently finely chopped using surgical scissors. The minced tissue fragments were incubated with 1% collagenase type IV at 37 °C and 200 rpm for 1 h. After the incubation, the tissue samples were vortexed and centrifuged at 1,700 rpm for 10 min. The resulting supernatants were removed, and the remaining cell pellet was resuspended in Dulbecco’s modified Eagle medium (DMEM) supplemented with 1% penicillin–streptomycin (p/s; 15140-122, Invitrogen) and 10% fetal bovine serum (FBS; 16000-044, Invitrogen) to culture the cells. The Ms-FLSs were then cultured at 37 °C in a humidified atmosphere of 5% CO_2_/95% air. All FLSs were cultured in DMEM (11995-065, Gibco) supplemented with 1% p/s and 10% FBS and maintained at 37 °C in a humidified incubator with 5% CO_2_ and 95% air. Upon reaching approximately 80% confluence, the culture medium was aspirated, and the cells were washed with DPBS (LB001-02, Welgene). The cells were subsequently treated with trypsin–EDTA for 1 min (Ms-FLSs) or 5 min (RA-FLSs and OA-FLSs) to detach the cells. All FLSs were used within five passages for the experiments.

### Osteoclast differentiation protocols

#### Bone marrow macrophages

Bone marrow was flushed from the femurs and tibias of C57BL/6N mice using alpha-modified Eagle’s medium (α-MEM) supplemented with 1% p/s and 10% FBS. After the removal of erythrocytes using a hypotonic buffer, the cells were cultured in α-MEM supplemented with 1% p/s and 10% FBS for 24 h. Nonadherent cells were then collected and subsequently cultured on Petri dishes in α-MEM supplemented with 1% p/s, 10% FBS and M-CSF for an additional 3 days. Unattached cells were removed by suction, and the remaining adherent cells were collected and used as bone marrow macrophages (BMMs). After this initial period, the cells were further differentiated by the addition of both M-CSF and RANKL for an additional 4 days. Similar to Raw264.7 cells, the culture medium of BMMs was replaced every 2 days.

#### Raw264.7 cells

Raw264.7 cells were differentiated into osteoclasts by culturing them in α-MEM supplemented with 1% p/s, 10% FBS, M-CSF (315-02, PeproTech, 30 ng/ml) and RANKL (390-TN-010/CF, R&D Systems) for 7 days. The culture medium was replaced every 2 days.

### Fluorescence-activated cell sorting

For the fluorescence-activated cell sorting (FACS) analysis, Ms-FLSs were separated into single cells in DPBS supplemented with 1% FBS. An antibody specific for an FLS marker, mouse anti-CD90.2 (14-0903-82, Invitrogen), was administered to primary cultured FLSs for 30 min at 4 °C to identify FLSs. The cells were washed three times with DPBS, centrifuged at 1,500 rpm for 5 min and incubated with a fluorescein isothiocyanate (FITC)-conjugated anti-rabbit immunoglobulin G (IgG) antibody for 30 min at 4 °C. After washes with DPBS, the centrifuged cells were suspended in DPBS supplemented with 1% FBS and passed through a 35-μm filter into a tube. The cell population was analyzed using a flow cytometer (BD LSRII, BD Biosciences), and the data were analyzed using FlowJo software (FlowJo). The population of CD90.2-positive cells was determined by comparing FITC-fluorescent dots with the negative control.

### RT‒qPCR

Total RNA was extracted from the cells using the Ribo^Ex^ extraction system (GeneAll) according to the manufacturer’s instructions. RNA was quantified using an ND-1000 spectrophotometer (Thermo Fisher Scientific). Complementary DNA was then synthesized using Accupower RocketScript Cycle RT PreMix (Bioneer) according to the manufacturer’s protocol. Reverse-transcription quantitative polymerase chain reaction (RT‒qPCR) was performed using PowerUp SYBR Green Master Mix (#A25741, Applied Biosystems) on a QuantStudio 3 RT‒PCR system (#A28567, Applied Biosystems) with the primers listed in Table 1. The RT‒qPCR cycling conditions were as follows: initial uracil DNA glycosylase activation at 50 °C for 2 min, followed by dual-lock DNA polymerase activation at 95 °C for 2 min. These steps were followed by denaturation at 95 °C for 15 s, annealing at 55 °C for 15 s and elongation at 72 °C for 1 min.

**Table 1:** Primers.

Human *OPG*	Forward	GTC AAG CAG GAG TGC AAT CG
	Reverse	TAG CGC CCT TCC TTG CAT T
Human *RANKL*	Forward	GGT GGA TGG CTC ATG GTT AGA T
	Reverse	GAG CAA AAG GCT GAG CTT CAA G
Human *M-CSF*	Forward	CGA GGA GGT GTC GGA GTA CTG T
	Reverse	AAT CAG CCG CTG CAG AGA CT
Human *CaSR*	Forward	ACA TTC CCC AGG TCA GTT ATG C
	Reverse	GAT GGT TCG GAG GAA AGA CTT G
Mouse *M-csf*	Forward	TGCCCGACTTCCCGTAAA
	Reverse	GCCTCCTCTTCCCGTCTTTT
Mouse *Tnf*	Forward	CAA GCC TGT AGC CCA TGT TGT
	Reverse	TTG GCC AGG AGG GCA TT
Mouse *Il-1β*	Forward	CTACAGGCTCCGAGATGAACAAC
	Reverse	TCCATTGAGGTGGAGAGCTTTC
Mouse *Il-6*	Forward	AGTTGCCTTCTTGGGACTGATG
	Reverse	GGGAGTGGTATCCTCTGTGAAGTCT
Mouse *Il-8*	Forward	TGCGTATCCTGCCTCAGACTT
	Reverse	CCCCATGTGGGCCTTAAAG
Human *SLC4A4*	Forward	ACA ATG ATG ATG AGA AAG ATC ACC A
	Reverse	ACT TGG CAT ACC GAG TGA CTG
Human *SLC4A7*	Forward	AAT TCC TAC GGG TGC TGA GG
	Reverse	GTA AGG AGG ACA GCA GGA GC
Human *SLC4A10*	Forward	GGT GCT TAT TCC AGA GGC GT
	Reverse	ATT ATC CGT AGG CAG CAG CG

### Transwell membrane migration assay

The migration assay was conducted using a transwell-polycarbonate membrane (6.5-mm insert, 8.0-μm pore size). The inserts were filled with 200 μl of RA-FLSs or Ms-FLSs (5 × 10^4^ cells per well, Cell Applications) containing 1% FBS and reagents. TNF and M-CSF + RANKL (M + R) dissolved in 500 μl of DMEM or α-MEM at different pH values were added to the bottom chamber, along with conditioned media from mature osteoclasts cultured on dentin discs, which were stimulated by Raw264.7 cells and BMMs using five distinct FLS-conditioned media. The cells were incubated for 6 h. Thereafter, the DMEM in the bottom chamber was removed, chilled methanol was added and the inserted membranes were soaked for 1 min at −20 °C. The chilled methanol was removed, and the plate was washed three times with DPBS. A 4′,6-diamidino-2-phenylindole (DAPI) solution was mixed with distilled water and loaded into the bottom chamber. The plates were then incubated for 30 min in the dark. The medium was carefully removed from the insert, and distilled water was added to the bottom chamber at room temperature. DAPI fluorescence was measured at 405 nm using an LSM 700 Zeiss confocal microscope (Fluoview, Carl Zeiss). FLS migration was determined on the basis of the number of nuclei stained with DAPI on the transwell membrane.

### Immunostaining of the insert membrane in a transwell culture system

After the migration assay, the inserts were prepared for immunostaining. The membrane of each insert was carefully cut out, isolated and immunostained with an NBCn1 antibody (ab82335, Abcam). The isolated membranes were first placed in a 100 mM glycine solution for 10 min at 4 °C, followed by an incubation with 0.5% bovine serum albumin (BSA) for 1 h at room temperature in the dark. The membranes were then incubated overnight at 4 °C with the primary antibody diluted 1:100 and subsequently incubated with the secondary antibody, rhodamine-tagged goat anti-rabbit IgG, for 1 h at room temperature. Finally, the membranes were mounted on glass slides using Fluoromount-G containing DAPI (Electron Microscopy Sciences). Fluorescence images were acquired using an LSM 700 Zeiss confocal microscope (Fluoview) and analyzed with ZEN (Zeiss analysis software).

### MTT assay

Raw264.7 cells, BMMs, Ms-FLSs and RA-FLSs (1 × 10^4^ cells per well) were seeded in 96-well plates and treated with S0859 or CaCl_2_ for 24 h. Tetrazolium bromide dye (MTT; 298-93-1, 2 mg/ml; Merck) was mixed with heated DPBS (37 °C). After the addition of the MTT solution, the cells were incubated in the dark at 37 °C in a humidified atmosphere of 5% CO_2_ and 95% air for 2 h. The supernatant was carefully removed from the plates, and dimethyl sulfoxide (100%) was added to the plates. The absorbance was measured at 570 nm using a fluorescence microplate reader (Varioskan LUX, Thermo Fisher Scientific).

### Measurement of sodium-bicarbonate cotransporter (NBC) activity

2′-7′-Bis-(carboxyethyl)-5-(and-6)-carboxyfluorescein (BCECF-AM; B1170, Invitrogen) was used to measure intracellular pH changes in RA-FLSs, Raw264.7 cells and BMMs at dual excitation wavelengths (440/495 nm) and a single emission wavelength (530 nm). 5-(*N*-ethyl-*N*-isopropyl) amiloride (EIPA; A3085, Sigma), an inhibitor of the major pH modulator sodium/hydrogen exchanger, was mixed with the HCO_3_^−^ solution to measure only the NBC activity-mediated pH modulation. RA-FLSs, Raw264.7 cells and BMMs were treated with CaCl_2_, M-CSF, RANKL and S0859 and loaded onto coverslips with 0.1% pluronic F-127 and 20 μM BCECF-AM perfused with a physiological salt solution (the composition was previously described in ref. ^14^ and was called regular solution). NBC activity was measured by incubating the cells with CO_2_-saturated HCO_3_^−^-buffered medium with 10 μM EIPA and then acidifying the cells with sodium-free bicarbonate-buffered medium. The emitted fluorescence was monitored using a CCD camera (Photometrics) attached to an inverted microscope (Olympus) and analyzed using a MetaFluor system (Molecular Devices). All BCECF fluorescence images were obtained at 1-s intervals, and the background fluorescence was subtracted from the raw background signals at each wavelength.

### Immunofluorescence staining and confocal imaging

Raw264.7 cells and BMMs seeded onto cover glasses were treated with M-CSF and RANKL for 24 h. The cells were fixed with chilled methanol (−20 °C) for 10 min, a 100 mM glycine solution was added and incubated for 10 min at 4 °C, and cells were washed three times with cold DPBS. The cells were then blocked with 0.5% BSA in DPBS supplemented with 10% goat serum for 1 h at room temperature in the dark, incubated with NBCn1 and NFATc1 (sc-7294; SANTACRUZ Biotechnology) antibodies at 4 °C overnight, and washed three times with 0.5% BSA in DPBS. The cells were treated with a secondary rhodamine-tagged goat anti-rabbit IgG antibody for 1 h at room temperature to detect the primary antibody. After the incubation, the cells were washed three times with DPBS, and the cover glasses were carefully attached and mounted on a glass slide with DAPI-containing Fluoromount-G. Fluorescence images were acquired using a Zeiss LSM700 confocal microscope (Fluoview, Carl Zeiss) and analyzed using ZEN (Carl Zeiss) software.

### CIA mouse model

All experimental animal procedures were performed in accordance with the Gachon University guidelines and approved by the Animal Care and Use Committee of Gachon University (LCDI-2022-0118, LCDI-2022-0039 and LCDI-2024-0029). DBA/1 (6-week-old male, 22–25 g) mice were purchased from KOATECH and Orient Bio. The CIA model was induced by administering 4 μg of bovine type II collagen per gram of body weight (20021, Chondrex) dissolved in complete Freund’s adjuvant (F5881, Sigma) at a 1:1 ratio via intradermal injection for the first immunization. After 2 weeks, 4 μg of bovine type II collagen per gram of body weight was dissolved in incomplete Freund’s adjuvant (F5506, Sigma) at a 1:1 ratio and administered as a booster injection. The animals were divided into the CIA, CIA + S0859, CIA + adalimumab, and control (vehicle) groups. At the 4-week mark, adalimumab (HY-P9908A, MCE) was administered via a tail vein injection at a dosage of 0.25 mg/kg (body weight). Concurrently, S0859 was dissolved in dimethyl sulfoxide (19128, Bio Life Solutions), diluted with saline and injected into the tail vein at a concentration of 0.8 mg/kg (body weight). Two weeks after the initial administration of adalimumab and S0859, a second dose was administered at the same concentration. Sixty mice were divided equally into control and vehicle groups, and 35 mice were used for CIA modeling. The mice in the experimental and control groups were euthanized 6 weeks after the second S0859 injection. The serum, femurs and paws of the animals in all the groups were collected for analysis. Changes in body weight, the paw score and paw thickness were measured every 2 weeks, and paw images were obtained.

### Determination of the paw score and thickness

Scoring was performed in a blinded manner to estimate the severity of CIA in mouse paws, which ranged from 0 to 4 for each paw^12^: normal paw, 0; one inflamed and swollen toe, 1; more than one inflamed and swollen toe, 2; the entire paw was inflamed and swollen, 3; and severe inflammation and swelling of the ankylosed paw, 4 (relatively extended range, 0–16). The paw score was measured by three independent and blinded observers, and the thickness of the hind paw was measured using calipers.

### Three-dimensional micro-CT

The dissected femurs from all the groups were fixed with 10% neutral-buffered formalin (FR2013-100-00, Biosesang for 1 h. Micro-computed tomography (CT) was conducted on the joint of the femurs using a micro-CT scanner (SKYSCAN 1276, Bruker) under the following conditions: exposure at 50 kV, 200 μA, 0.25 mm artificial intelligence (AI) filter, 200 μm offset and 1.7 mm analysis height.

### Osteoclast stimulation with FLS medium

FLSs isolated from 6-week-old mice were cultured in 24-well plates (5 × 10^4^ cells per well) and stimulated with TNF for 6 h. Raw264.7 cells and BMMs (1 × 10^4^ cells per well) were treated with FLS-conditioned medium and then incubated at 37 °C for 4–7 days.

### TRAP staining

Raw264.7 cells and BMMs (1 × 10^4^ cells per well) were seeded in a 96-well plate and incubated at 37 °C to allow cells to adhere to the bottom of the well. Raw264.7 cells were then cultured in medium containing M-CSF and RANKL for 7 days. BMMs were first cultured in medium containing M-CSF for 3 days, followed by an additional 4 days in medium containing both M-CSF and RANKL. Thereafter, the cells were cultured in differentiation medium containing RANKL, M-CSF and FLS medium (α-MEM with 1% p/s and 10% FBS). After the incubation, the cells were fixed with a 4% formaldehyde solution (Tech & Innovation) for 10 min. The dissected paws were fixed with 10% neutral-buffered formalin for 1 h, and the skin was removed. The fixed joints were decalcified in Rapid Cal Immuno (6040; BBC Biochemical) for 2 weeks. Tissues were embedded in paraffin and placed on slides. Thereafter, the fixed cells and tissues were stained with a tartrate-resistant acid phosphatase (TRAP)/alkaline phosphatase staining kit (FUJIFILM) according to the manufacturer’s instructions. Images of the stained cells and paws were obtained using a Nikon DIAPHOT 300 inverted optical microscope (Nikon) equipped with a KOPIC HK6E3 digital camera (SONY). The intensity and number of stained cells were analyzed using MetaMorph software (Molecular Devices). For TRAP staining of bone slices, the numbers of osteoclasts/bone perimeters and osteoclast surface/bone surface were analyzed using Osteo-measure software (Osteometrics).

### Soluble TRAP measurement

Raw264.7 cells were cultured for 7 days. Thereafter, the culture medium was collected and stored on ice. An enzymatic analysis was performed in a 96-well microplate. Each sample of medium (30 μl) was added to the plate on ice, followed by the addition of 30 μl of soluble TRAP analysis buffer (95 mM sodium acetate, 47.5 mM sodium tartrate and 5 mg/ml *p*-nitrophenyl phosphate, pH 4.5). The cells were then incubated at 37 °C for 30 min. The plate was immediately transferred onto ice, and 60 μl of cold 0.5 N NaOH was added to each well to terminate the reaction. The absorbance of the reaction mixture was measured at 405 nm using a VARIOSKAN LUX ELISA reader (Thermo Fisher Scientific).

### Pit assay

Raw264.7 cells (1 × 10^4^ cells per well) were seeded into plates with an OsteoSite dentin disc (Immunodiagnosticsystems, United Kingdom) and cultured for 7 days in differentiation medium containing RANKL, M-CSF and FLS medium (α-MEM with 1% p/s and 10% FBS). BMMs (1 × 10^4^ cells per well) were initially treated with M-CSF for 3 days, followed by an additional 4 days of culture in differentiation medium containing RANKL, M-CSF and FLS medium (α-MEM with 1% p/s and 10% FBS). After 7 days of culture, the mature osteoclasts in each well were removed using a 5% sodium hypochlorite solution and washed three times with distilled water. The resorption areas on the dentin surface were captured using a Nikon DIAPHOT 300 inverted optical microscope (Nikon) equipped with a KOPIC HK6E3 digital camera (SONY). The total area and absorption area of each image were calculated using MetaMorph software (Molecular Devices).

### Western blotting

Raw264.7 cells and BMMs (2 × 10^5^ cells per well) were seeded into a six-well plate and incubated at 37 °C to allow cells to adhere to the bottom of the wells. Raw264.7 cells were then cultured in medium containing M-CSF and RANKL for either 3 or 7 days. BMMs were initially cultured in medium containing M-CSF for 3 days, followed by an additional 4 days of culture in medium containing both M-CSF and RANKL. After these initial treatments, the cells were exposed to differentiation medium containing RANKL, M-CSF and FLS medium (α-MEM with 1% p/s and 10% FBS). Proteins were isolated from the cells using a lysis buffer containing 20 mM Tris, 150 mM NaCl, 2 mM EDTA, 1% Triton X-100 and a protease inhibitor mixture. The protein concentration was quantified using the Bradford assay (5000207, Quick Start BSA standard, Bio-Rad). Proteins were then denatured in SDS sample buffer at 37 °C for 30 min. Denatured protein samples (30 μg each) were subjected to SDS‒PAGE. Proteins were visualized with NFATc1 (sc-7294, SANTACRUZ Biotechnology), NBCn1 and β-actin (A3854, Sigma) antibodies with an enhanced chemiluminescence solution (Thermo Fisher Scientific). Images were obtained using an Amersham ImageQuant 800 imaging system (29399481, Cytiva).

### ELISA

The levels of M-CSF (MMC00B, R&D Systems) and C-terminal telopeptide of type I collagen (CTX-1; AC-06F1, Immunodiagnosticsystems) in the cell culture supernatants were quantified using their respective enzyme-linked immunosorbent assay (ELISA) kits, according to the protocols provided by the manufacturers.

### Statistical analysis

The experimental results are presented as the mean ± standard error of the mean (s.e.m.). Differences between the mean values of two samples were analyzed using analysis of variance (ANOVA). The significance of differences in each experiment was determined using ANOVA. Significance is indicated by an asterisk for comparison with the control group and a number sign for comparison with the CIA group.

## Results

### Cytokine- and high-Ca^2+^-mediated FLSs activation induces the expression of bone destruction factors and NBC activation

**Fig. 1:**
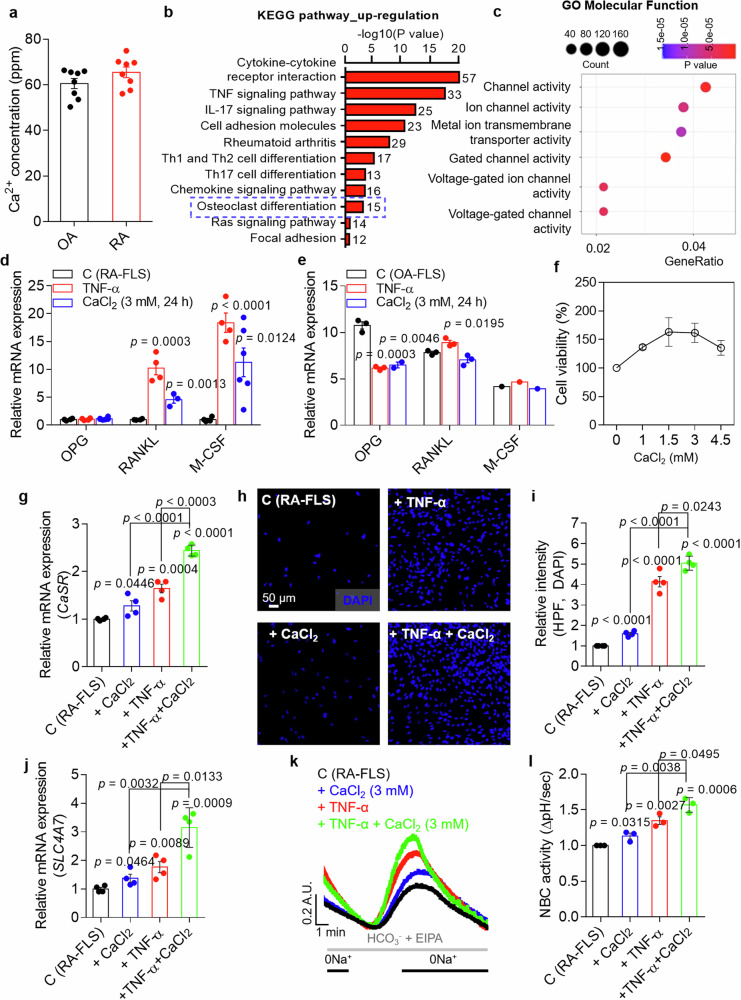
Cytokine- and high-Ca^2+^-mediated FLSs activation induces the expression of bone destruction factors and NBC activation. **a** Measurement of the Ca^2+^ concentration in the synovial fluid of patients with OA or RA. **b** KEGG pathway analysis of genes with upregulated expression in TNF-treated human RA-FLSs. **c** Significant changes in the expression of ion channel and transporter genes in TNF-treated human RA-FLSs were observed on the basis of the enriched GO terms for molecular function. **d** Relative mRNA expression levels (*OPG*, *RANKL* and *M-CSF*) in human RA-FLSs treated with TNF or 3 mM CaCl_2_ for 24 h. The bars represent the mean ± s.e.m. (*n* = 3–6). **e** Relative mRNA expression levels (*OPG*, *RANKL* and *M-CSF*) in human OA-FLSs treated with TNF or 3 mM CaCl_2_ for 24 h. The bars represent the mean ± s.e.m. (*n* = 2–3). **f** The viability of human RA-FLSs treated with different doses of CaCl_2_ (0, 1, 1.5, 3 or 4.5 mM) for 24 h (*n* = 3). **g** The mRNA expression levels of the *CaSR* in human RA-FLSs cultured under the indicated conditions for 24 h. The bars represent the mean ± s.e.m. (*n* = 4). **h** A migration assay of human RA-FLSs cultured under the indicated conditions for 6 h. Fluorescence staining with DAPI (blue). Scale bar, 50 μm. **i** An analysis of the total DAPI intensity to examine human RA-FLS migration. The bars represent the mean ± s.e.m. (*n* = 4). **j** The mRNA expression level of *SLC4A7* in human RA-FLSs cultured under the indicated conditions for 24 h. The bars represent the mean ± s.e.m. (*n* = 4). **k** NBC activity was assessed by measuring changes in the pH_i_ values of human RA-FLSs cultured under the indicated conditions for 24 h. **l** An analysis of the NBC activity of human RA-FLSs cultured under the indicated conditions for 24 h. The bars represent the mean ± s.e.m. (*n* = 3).

Patients with RA exhibit features of bone and cartilage erosion. Thus, we hypothesized that Ca^2+^ released from destroyed bones increases synovial Ca^2+^ levels. Compared with those of patients with OA, the synovial fluids of patients with RA presented mildly increased Ca^2+^ levels; however, no statistically significant difference was found (Fig. 1a; the standard curve for the Ca^2+^ concentration is shown in Supplementary Fig. 1). The synovial Ca^2+^ concentration was approximately 60 ppm, which equates to 1.5 mM. Several studies have addressed the robust expression of cytokines and chemokines in inflamed tissues, including RA joints^15^. Thus, we verified the differential gene expression levels in FLSs between the control group and group treated with RA-associated inflammatory cytokine TNF. The expression of genes related to cytokine signaling, chemokine signaling, adhesion, T cell differentiation and osteoclastogenesis was increased in TNF-treated human RA-FLSs (Fig. 1b). We focused on the upregulated osteoclastogenesis-related gene M-CSF (Supplementary Fig. 2). The GO enrichment analysis revealed that treatment with TNF induced significant changes in the expression of ion channel- and transporter-related genes (Fig. 1c). The Ca^2+^ concentration in the bone marrow markedly varies from 0.1 to 4.5 mM (ref. ^16^). In this study, cells were stimulated with 3 mM extracellular Ca^2+^ for high Ca^2+^ stimulation, and the levels of factors involved in osteoclastogenesis were examined in the presence of inflammatory cytokines and increased extracellular Ca^2+^ levels. Stimulation with TNF and 3 mM Ca^2+^ increased the mRNA expression of *RANKL* and *M-CSF*, but not that of osteoprotegerin (*OPG*), in human RA-FLSs (Fig. 1d). In human OA-FLSs, stimulation with TNF and 3 mM Ca^2+^ did not increase the expression of these genes (Fig. 1e). Increased Ca^2+^ levels did not attenuate cell viability or mildly induce cellular proliferation (Fig. 1f). Extracellular Ca^2+^ is sensed by the Ca^2+^-sensing receptor (CaSR), which is expressed in epithelial cells and is involved in various cellular functions, such as differentiation, proliferation and bone metastatic homeostasis^17^. The activation of CaSR induces an increase in the intracellular Ca^2+^ concentration, ERK1/2 activation and cellular proliferation^8^. Notably, stimulation with TNF and 3 mM Ca^2+^ increased the expression of the *CaSR* mRNA in human RA-FLSs (Fig. 1g). According to our previous study, inflammatory cytokines enhance FLS migration^12^. Stimulation with TNF and 3 mM Ca^2+^ in the extracellular medium increased human FLS migration (Fig. 1h, i). Ca^2+^-mediated ERK1/2 activation is assumed to promote NBC activity^18^; therefore, we verified the expression and activity of a migration-related module, the NBC encoded by *SLC4A7*, in the presence of 3 mM Ca^2+^. An increase in *SLC4A7* mRNA expression was induced by 3 mM Ca^2+^ in human RA-FLSs (Fig. 1j). In addition, NBC activity was increased by treatment with 3 mM Ca^2+^ in human RA-FLSs (Fig. 1k, l). Costimulation with TNF and 3 mM Ca^2+^ had additive effects on the expression and activity of NBC in human RA-FLSs (Fig. 1j–l). These results indicate that cytokine- and extracellular high-Ca^2+^-mediated RA-FLS activation induces the expression of bone destruction factors and the migration-related module, NBCn1.

### Media from FLSs exposed to cytokines and high Ca^2+^ levels induce NFATc1 activation in osteoclasts

**Fig. 2:**
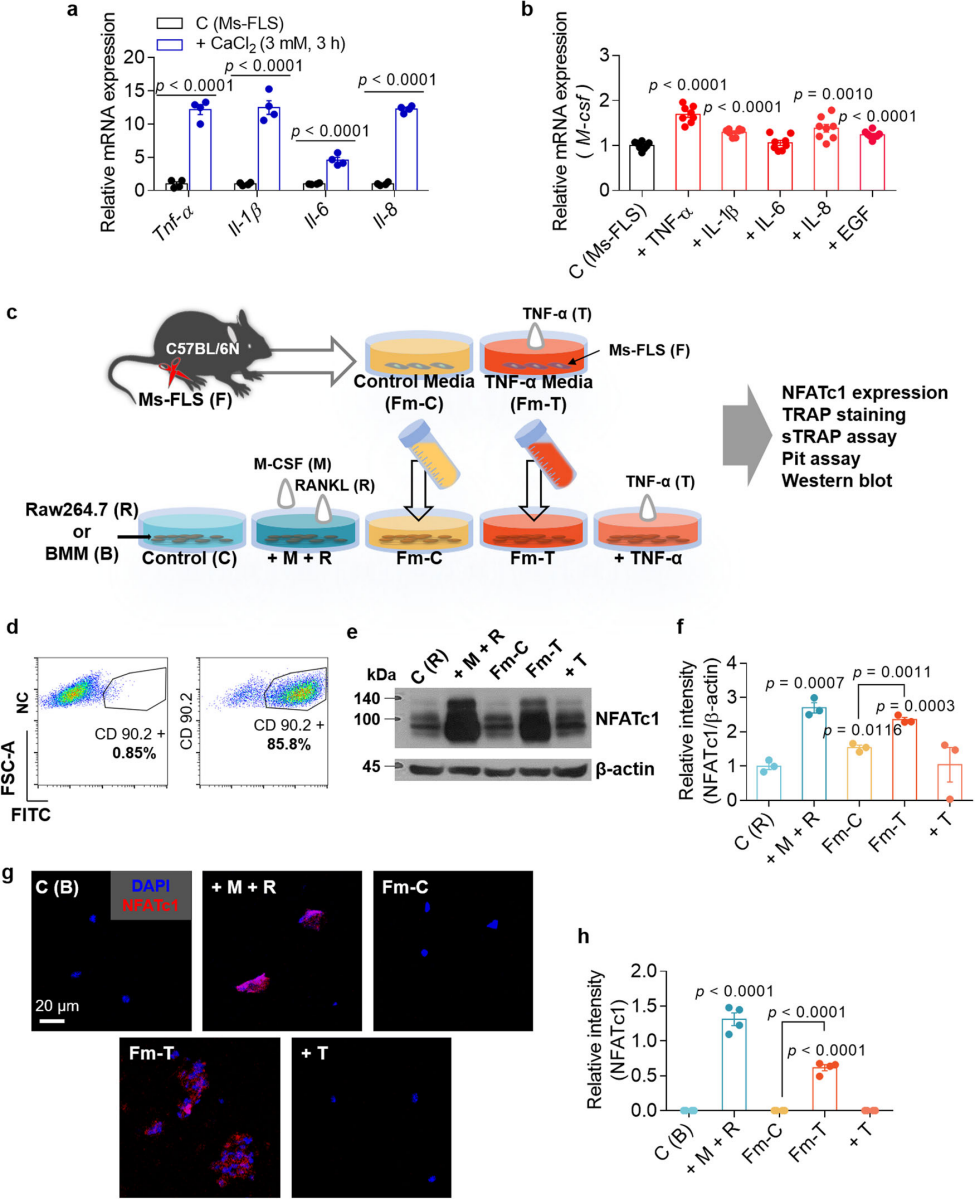
Medium from FLSs exposed to cytokines and high Ca^2+^ levels induces NFATc1 activation in osteoclasts. **a** Relative mRNA levels of inflammatory cytokines (*Tnf*, *Il-1β*, *Il-6* and *Il-8*) in isolated primary Ms-FLSs treated with 3 mM CaCl_2_ for 3 h. The bars represent the mean ± s.e.m. (*n* = 4). **b** Relative mRNA levels of M-CSF in Ms-FLSs subjected to the indicated conditions (10 ng/ml TNF, IL-1β, IL-6, IL-8 and EGF) for 6 h. The bars represent the mean ± s.e.m. (*n* = 8). **c** A schematic of osteoclast maturation and subsequent assays conducted in the presence of the indicated stimuli. FLS medium without or with TNF is indicated as Fm-C or Fm-T, respectively. These media were administered to Raw264.7 cells and BMMs. **d** FACS scatter plots showing the CD90.2-positive cell population, marked as FITC-positive, among Ms-FLSs. N.C., negative control. **e** NFATc1 protein levels in Raw264.7 cells stimulated with the indicated cytokines for 3 days. β-Actin was used as a loading control. **f** An analysis of the band intensity of the NFATc1 protein in Raw264.7 cells. The bars represent the mean ± s.e.m. (*n* = 3). **g** Immunofluorescence staining for NFATc1 (red) and DAPI (blue) in BMMs subjected to the indicated conditions. Scale bar, 20 μm. **h** An analysis of the normalized intensity (total intensity/measuring area) of NFATc1 in cells cultured under the indicated conditions. The bars represent the mean ± s.e.m. (*n* = 4). F, FLSs; T, TNF; M, M-CSF; R, RANKL; C (R), Raw264.7; C (B), BMMs; C, control.

We determined the effects of high Ca^2+^ levels in FLSs on the expression of inflammatory cytokines, which were selected according to the major cytokines involved in RA. The experimental system was generated in mice to maintain the strain in subsequent experiments with isolated osteoclasts; thus, we isolated FLSs from the joints of the mice. We confirmed that treatment with 3 mM extracellular Ca^2+^ increased the mRNA expression of the inflammatory cytokines *Tnf, Il-1β*, *Il-6* and *Il-8* in isolated primary Ms-FLSs (Fig. 2a). Cytokine-exposed FLSs, but not IL-6-exposed FLSs, increased the expression of the M-CSF mRNA in Ms-FLSs (Fig. 2b). As a method to elucidate the cytokine feedback loops involved in RA, we designed and illustrated an experimental scheme to support the understanding of the experimental approach shown in Fig. 2c. Isolated mouse primary FLSs were stimulated with or without TNF. Isolated Ms-FLSs were verified by staining with an antibody against the FLS marker CD90.2 (ref. ^19^), and CD90.2(+) FLSs were used in subsequent experiments (Fig. 2d). Conditioned or stimulated FLS media were administered to Raw264.7 cells and primary BMMs. Notably, osteoclastogenesis requires factors involved in differentiation and proliferation, such as M-CSF and RANKL^11^. The expression of the master transcription factor nuclear factor of activated T cells (NFATc1)^11^ was determined to assess osteoclast differentiation. Raw264.7 cells treated with M-CSF and RANKL exhibited increased expression of the NFATc1 protein (Fig. 2e, f). Similarly, Raw264.7 cells treated with FLS medium containing TNF (Fm-T, termed activated FLS medium) exhibited increased expression of NFATc1 (Fig. 2e, f). Treatment with FLS control medium (Fm-C, termed no stimulation FLS medium) or TNF alone (T) slightly increased NFATc1 expression in Raw264.7 cells, but the difference was not statistically significant. The nuclear localization of NFATc1 was also increased in M-CSF- and RANKL-treated (+ M + R) and activated FLS medium-treated (Fm-T) BMMs (Fig. 2g, h). These results indicate that increased cytokines and Ca^2+^ levels induce increased M-CSF expression in FLSs and that TNF-exposed FLS media potentially contain cytokines and osteoclastogenic factors. Thus, osteoclasts exhibit increased NFATc1 activation in the presence of TNF-exposed FLS media, similar to the activation observed upon M-CSF + RANKL stimulation.

### Cytokine-exposed FLS medium stimulates osteoclasts maturation, which exhibits positive TRAP staining and increased resorption of the bone matrix

**Fig. 3:**
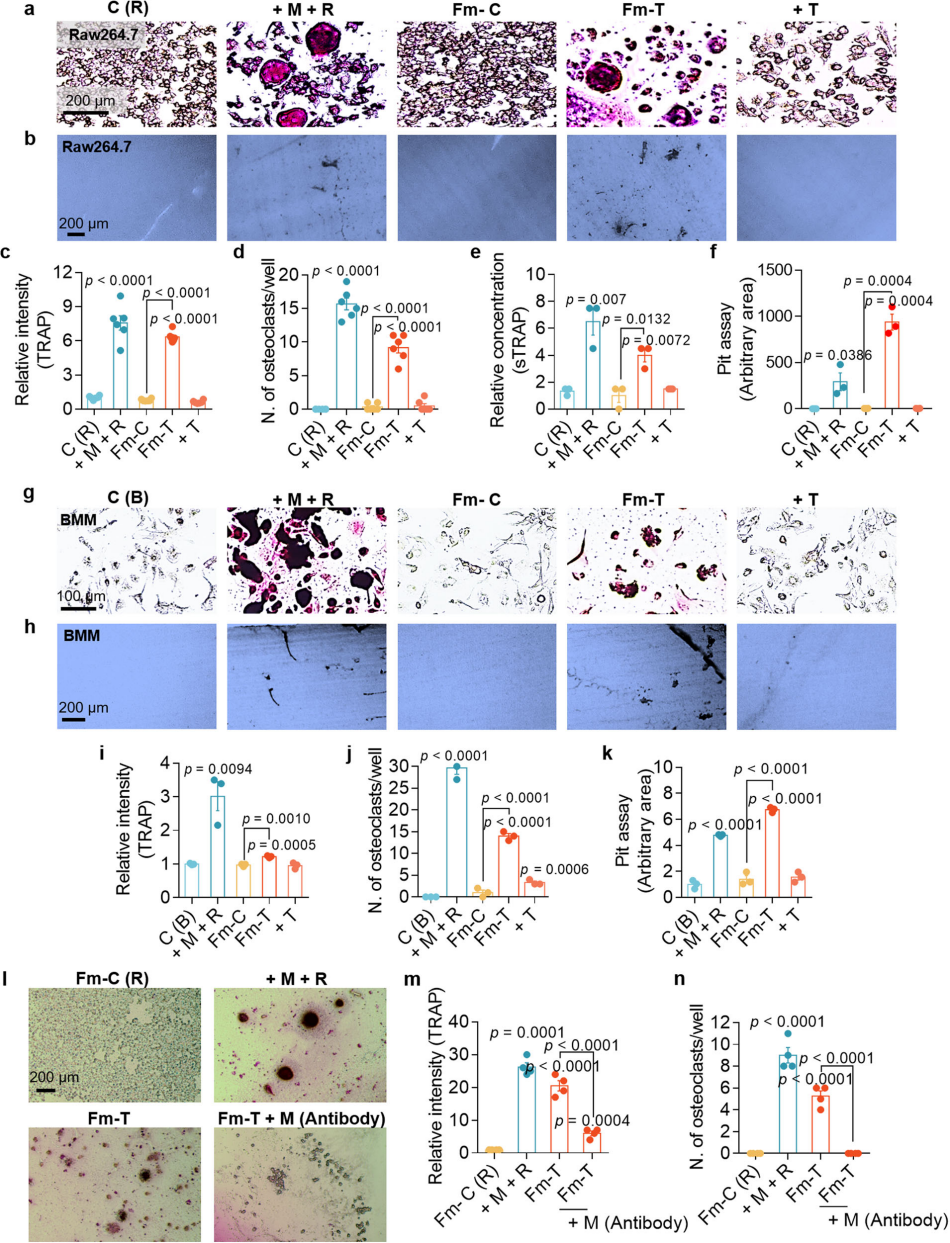
Cytokine-exposed FLS media stimulates osteoclasts maturation, which exhibits positive TRAP staining and increased resorption of the bone matrix. **a** Representative images of TRAP staining of osteoclasts derived from Raw264.7 cells subjected to the indicated conditions for 7 days. Scale bar, 200 μm. **b** Representative images of the bone resorption pit assay of Raw264.7 cells cultured with dentin discs and subjected to the indicated conditions for 7 days. Scale bar, 200 μm. **c** An analysis of the relative TRAP intensity in osteoclasts derived from Raw264.7 cells. The bars represent the mean ± s.e.m. (*n* = 6). **d** An analysis of the number of osteoclasts per well derived from Raw264.7 cells. The bars represent the mean ± s.e.m. (*n* = 6). **e** An analysis of the relative soluble TRAP (sTRAP) intensity in Raw264.7 cells. The bars represent the mean ± s.e.m. (*n* = 3). **f** An analysis of lacunae (dark area) formed by Raw264.7 cells. The bars represent the mean ± s.e.m. (*n* = 3). **g** Representative images of TRAP staining of osteoclasts derived from BMMs subjected to the indicated conditions. Scale bar, 100 μm. **h** Representative images of the bone resorption pit assay of BMMs cultured with dentin discs and subjected to the indicated conditions. Scale bar, 200 μm. **i** An analysis of the relative TRAP intensity in osteoclasts derived from BMMs. The bars represent the mean ± s.e.m. (*n* = 3). **j** An analysis of the number of osteoclasts per well derived from BMMs. The bars represent the mean ± s.e.m. (*n* = 3). **k** Analysis of lacunae (dark area) formed by BMMs. The bars represent the mean ± s.e.m. (*n* = 3). **l** Representative images of TRAP staining in osteoclasts derived from Raw264.7 cells treated with or without the M-CSF antibody and subjected to the indicated conditions for 7 days. Scale bar, 200 μm. **m** An analysis of the relative TRAP intensity in osteoclasts derived from Raw264.7 cells. The bars represent the mean ± s.e.m. (*n* = 4). **n** An analysis of the relative number of TRAP-positive osteoclasts derived from Raw264.7 cells. The bars represent the mean ± s.e.m. (*n* = 4).

Osteoclasts were incubated with M-CSF, RANKL and FLS medium to determine their osteoclastogenic activity. Osteoclastogenic activity, which represents multinucleated cells formation of osteoclasts, was determined via TRAP (osteoclast marker) staining and a pit assay, which verified the activity of bone resorption^20^. M-CSF + RANKL-treated (+ M + R) and activated FLS medium-treated (Fm-T) Raw264.7 cells were markedly positive for TRAP and presented an increased number of osteoclasts (Fig. 3a–d). Although the TNF-treated (+T) and control medium-treated (Fm-C) groups were also TRAP positive, the differences were not significant compared with those observed in the M + R- and Fm-T-treated groups. The M + R-treated group was used as a positive control for osteoclast activation. The level of the soluble form of TRAP was increased in the M + R-treated and Fm-T-treated groups (Fig. 3e). Notably, activated osteoclasts possess bone-absorbing functions. The pit area (bone-absorbing area) was increased in the M + R-treated and Fm-T-treated Raw264.7 cells (Fig. 3f). We confirmed TRAP staining and pit formation under the same conditions in BMMs. M + R- and Fm-T-treated BMMs were markedly TRAP positive, with increased numbers of osteoclasts (Fig. 3g–j) and pit areas (Fig. 3k). We assumed that the Fm-T group contained osteoclastogenic factors, such as M-CSF. Thus, the Fm-T group was incubated with an M-CSF antibody to remove the osteoclastogenic signals. The intensity and number of formed multinucleated cells of osteoclast were markedly reduced by treatment with the M-CSF antibody (Fig. 3l–n). These results indicate that cytokine-exposed FLS medium contains osteoclastogenic stimuli, which promote osteoclast activity and induce the formation of resorption pits.

### Components released from mature osteoclasts enhance FLS migration

**Fig. 4:**
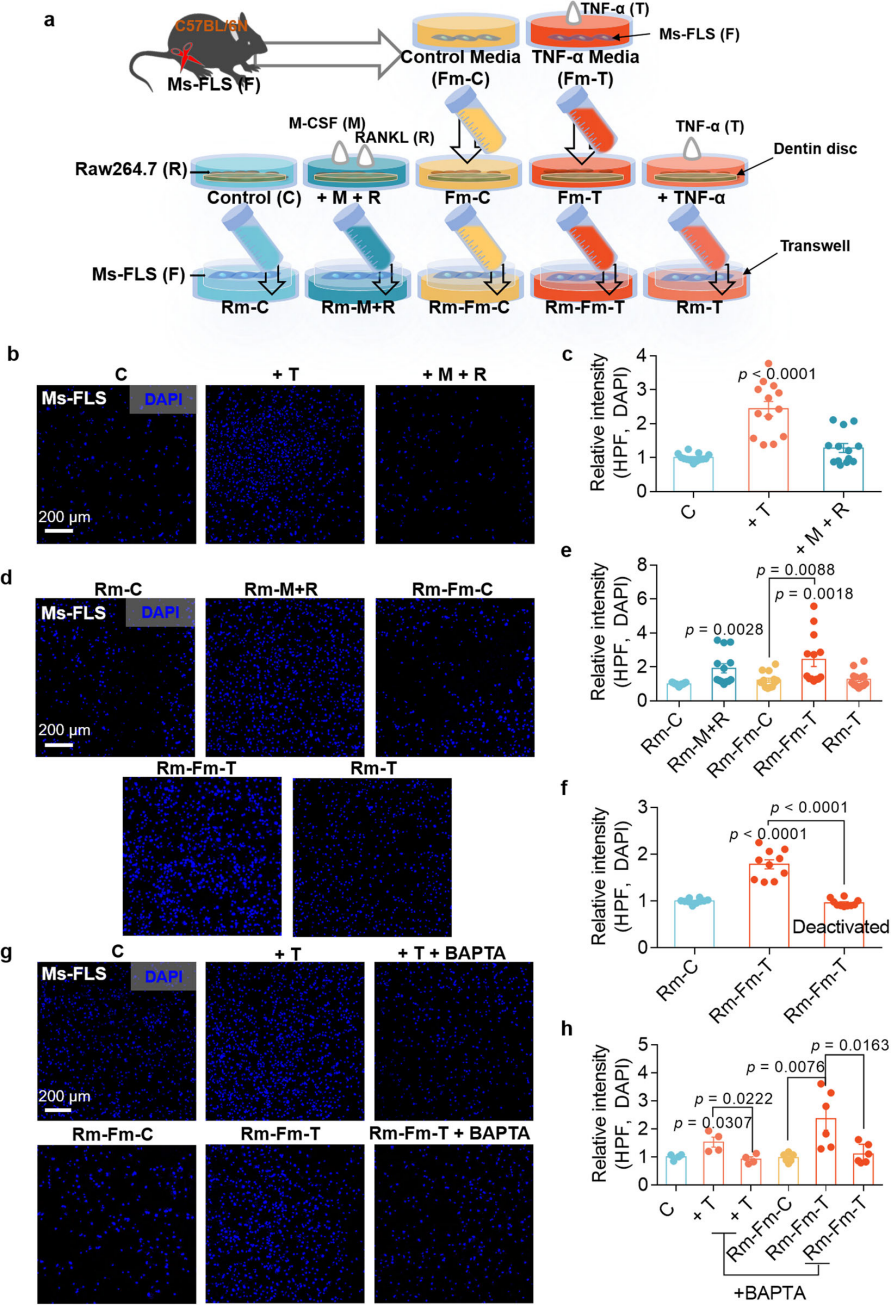
Components released from mature osteoclasts enhance FLS migration. **a** The schematic procedure of Ms-FLS migration cultured in the presence of the Raw264.7 cell medium-mediated stimuli. **b** Migration assays of isolated Ms-FLSs treated with TNF or M-CSF + RANKL for 6 h. Fluorescence staining with DAPI (blue). Scale bar, 200 μm. **c** An analysis of the total DAPI intensity was performed to assess Ms-FLS migration under the indicated conditions. The bars represent the mean ± s.e.m. (*n* = 13). **d** Migration assays of isolated Ms-FLSs subjected to the indicated conditions for 6 h. Fluorescence staining with DAPI (blue). Scale bar, 200 μm. **e** An analysis of the total DAPI intensity was performed to assess Ms-FLS migration under the indicated conditions. The bars represent the mean ± s.e.m. (*n* = 13). **f** Deactivated media were prepared from Rm-Fm-T (conditioned medium from Raw264.7 cells treated with FLS medium containing TNF). An analysis of the total DAPI intensity was performed to assess Ms-FLS migration in the presence or absence of deactivated media. The bars represent the mean ± s.e.m. (*n* = 10). **g** Migration assays of isolated Ms-FLSs subjected to the indicated conditions in the presence or absence of TNF and BAPTA for 6 h. Fluorescence staining with DAPI (blue). Scale bar, 200 μm. **h** An analysis of the total DAPI intensity was performed to assess Ms-FLS migration under the indicated conditions. The bars represent the mean ± s.e.m. (*n* = 6).

Mature osteoclasts possess bone resorption abilities. The cells were cultured in dentin disc-containing medium to verify bone resorption by osteoclasts. The dentin disc contains various components, including Ca^2+^, phosphorus and ions, such as Na^+^, K^+^ and Mg^2+^ (ref. ^21^). Thus, we determined the bone resorption activity of osteoclasts in dentin disc-containing medium with or without FLS-associated osteoclastogenic stimuli. We subsequently investigated whether the mobility of FLSs was affected by factors released by activated osteoclasts. We illustrated the experimental approach in Fig. 4a to provide a better understanding. FLSs were treated with M-CSF and RANKL to verify the effect of factors released by activated osteoclasts on FLS migration. FLSs exhibited increased migration in the presence of TNF, but their migratory ability was not affected by M-CSF + RANKL, suggesting that FLS migration was not affected by M-CSF + RANKL stimulation (Fig. 4b, c). Raw264.7 cells were exposed to five different FLS media and cultured for 8–9 days until they reached the mature state. New FLSs were exposed to dentin disc-containing Raw264.7 cells medium in the lower chamber of the transwell system to determine the effects of the factors released by mature osteoclasts on FLS migration. FLS migration was increased in the mature Raw264.7-conditioned medium after exposure to M-CSF + RANKL (termed Rm-M + R) and TNF (termed Rm-Fm-T) (Fig. 4d, e). Thus, we hypothesized that the biological factors released from Raw264.7 cells into the medium influence FLS migration. Briefly, the cells were treated with deactivated medium (termed deactivated Rm-Fm-T), which was boiled and chilled. Compared with Rm-Fm-T medium, deactivated Rm-Fm-T medium did not increase FLS migration (Fig. 4f and Supplementary Fig. 3). The dentin disc contains a Ca^2+^ component^22^. Therefore, to determine the effect of Ca^2+^ released from the dentin disc on FLS migration, the Ca^2+^-chelating drug BAPTA was added to remove Ca^2+^ from the dentin-extracted medium. The increase in FLS migration caused by the released Ca^2+^ in the Rm-Fm-T medium was inhibited by BAPTA treatment through the Ca^2+^ chelation (Fig. 4g, h). These results indicate that the components, including Ca^2+^, release from dentin-containing mature osteoclast medium increase FLS migration.

### Osteoclast activation reveals the enhanced expression of migratory module, NBC

**Fig. 5:**
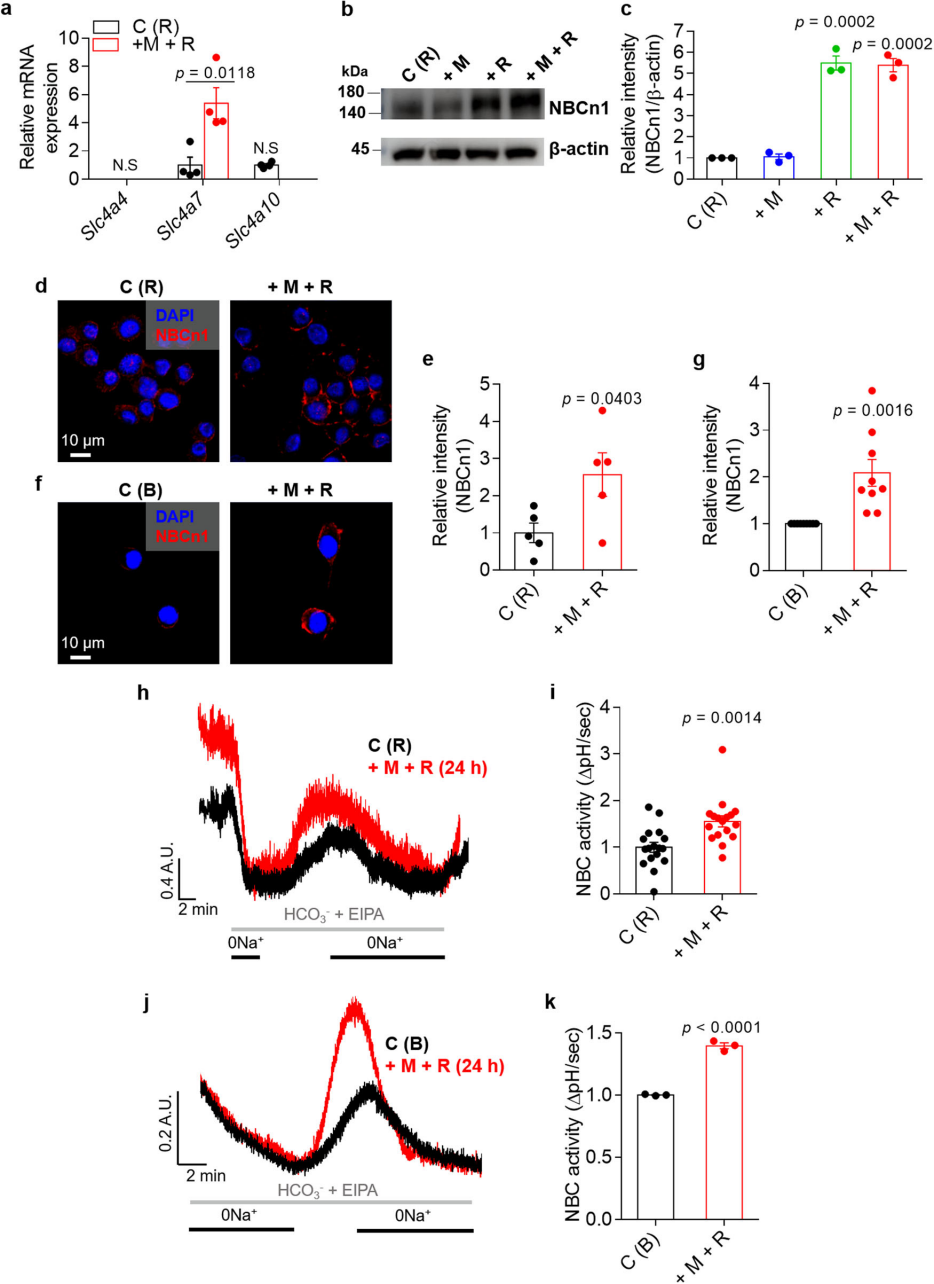
Osteoclast activation reveals the enhanced expression of migratory module, NBC. **a** Relative mRNA levels of *Slc4a4*, *Slc4a7* and *Slc4a10* in Raw264.7 cells treated with or without M-CSF and RANKL for 24 h. The bars represent the mean ± s.e.m. (*n* = 4). **b** NBCn1 protein levels in Raw264.7 cells treated with or without M-CSF and RANKL for 24 h. **c** An analysis of the NBCn1 intensity. The bars represent the mean ± s.e.m. (*n* = 3). **d**, **f** Immunofluorescence staining for NBCn1 (red) and DAPI (blue) in Raw264.7 cells (**d**) and BMMs (**f**) treated with or without M-CSF and RANKL for 24 h. Scale bar, 10 μm. **e**, **g** An analysis of the normalized intensity (total intensity/measuring area) of NBCn1 in Raw264.7 cells (**e**) and BMM (**g**) cells. The bars represent the mean ± s.e.m. (*n* = 5–9). **h** NBC activity was assessed by measuring the changes in the pH_i_ values of Raw264.7 cells stimulated with or without M-CSF and RANKL for 24 h. **i** An analysis of NBC activity in Raw264.7 cells (R). The bars represent the mean ± s.e.m. (*n* = 17). **j** NBC activity was assessed by measuring changes in the pH_i_ values of BMMs stimulated with or without M-CSF and RANKL for 24 h. **k** An analysis of NBC activity in BMMs under the indicated conditions. The bars represent the mean ± s.e.m. (*n* = 3).

Previously, we reported that the migratory function of FLSs and the severity of RA in CIA-induced mice were attenuated by NBC inhibition^12^. We hypothesized that osteoclast fusion and maturation are modulated by the migratory module to facilitate fusion and migration. We verified the role of the migration-related protein in osteoclasts by measuring the expression of the *Slc4a* family gene set encoding NBC, *Slc4a4*, *Slc4a7* and *Slc4a10*, in Raw264.7 cells. Treatment with M-CSF and RANKL increased the expression of the *Slc4a7* mRNA (Fig. 5a). The protein expression of NBCn1, which is encoded by *Slc4a7*, was also increased in the presence of RANKL (Fig. 5b, c). NBCn1 is a plasma-membrane-associated protein^23^. Increased membrane expression of NBCn1 was observed in Raw264.7 cells and BMMs in the presence of M-CSF and RANKL (Fig. 5d–g). The increased expression of the NBCn1 protein reflects increased NBC activity. NBC activity was also increased in Raw264.7 cells and BMMs after treatment with M-CSF + RANKL (Fig. 5h–k). These results indicate that osteoclast activation by M-CSF and RANKL increases NBC expression and activity.

### NBC inhibition attenuates osteoclastogenesis and bone resorption in Raw264.7 cells

**Fig. 6:**
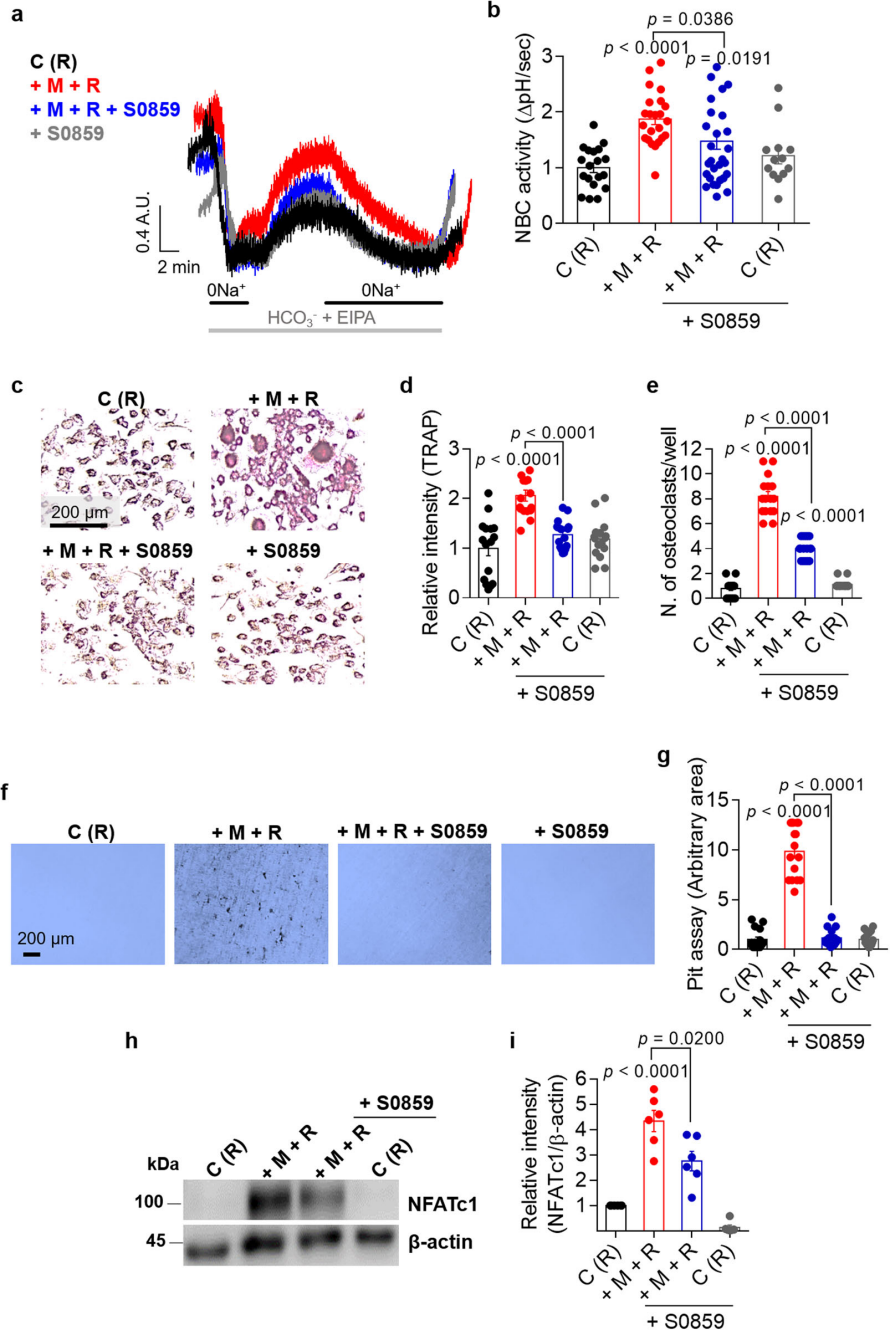
NBC inhibition attenuates osteoclastogenesis and bone resorption in Raw264.7 cells. **a** NBC activity was assessed by measuring the changes in the pH_i_ values of M-CSF- and RANKL-stimulated Raw264.7 cells treated with or without 20 μΜ S0859 for 24 h. **b** An analysis of NBC activity in Raw264.7 cells (R). The bars represent the mean ± s.e.m. (*n* = 13–30). **c** Representative images of TRAP staining in Raw264.7 cells subjected to the indicated conditions in the presence or absence of 20 μΜ S0859 for 7 days. Scale bar, 200 μm. **d** An analysis of the relative TRAP intensity in osteoclasts derived from Raw264.7 cells subjected to the indicated conditions for 7 days. The bars represent the mean ± s.e.m. (*n* = 16). **e** An analysis of the number of osteoclasts derived from Raw264.7 cells subjected to the indicated conditions for 7 days. The bars represent the mean ± s.e.m. (*n* = 16). **f** Bone resorption pit assay of Raw264.7 cells cultured with dentin discs and subjected to the indicated conditions in the presence or absence of 20 μΜ S0859 for 7 days. Scale bar, 200 μm. **g** An analysis of lacunae (dark areas) formed by Raw264.7 cells. The bars represent the mean ± s.e.m. (*n* = 16). **h** NFATc1 protein levels in Raw264.7 cells exposed to the indicated stimuli for 7 days. β-Actin was used as a loading control. **i** An analysis of the band intensity of the NFATc1 protein in Raw264.7 cells. The bars represent the mean ± s.e.m. (*n* = 6).

NBC activity was measured in Raw264.7 cells treated with or without the NBC inhibitor S0859 to determine the role of NBC in osteoclast activation^12^. Treatment with S0859 attenuated M-CSF + RANKL-mediated NBC activity in Raw264.7 cells (Fig. 6a, b). Treatment with 40 μM S0859 did not affect the viability of Raw264.7 cells (Supplementary Fig. 4a). We determined whether the attenuated NBC activity of Raw264.7 cells induces cellular fusion and bone resorption. Treatment with S0859 markedly attenuated M + R-stimulated multinucleated cell fusion and decreased the number of mature osteoclasts (Fig. 6c–e) and bone resorption in Raw264.7 cells (Fig. 6f, g). NFATc1 protein expression in Raw264.7 cells treated with or without S0859 was evaluated to determine the master transcriptional regulator of osteoclastogenesis. M-CSF + RANKL-treated Raw264.7 cells expressed NFATc1; however, treatment with S0859 reduced NFATc1 protein expression (Fig. 6h, i).

### NBC inhibition attenuates osteoclastogenesis and bone resorption in BMMs

**Fig. 7:**
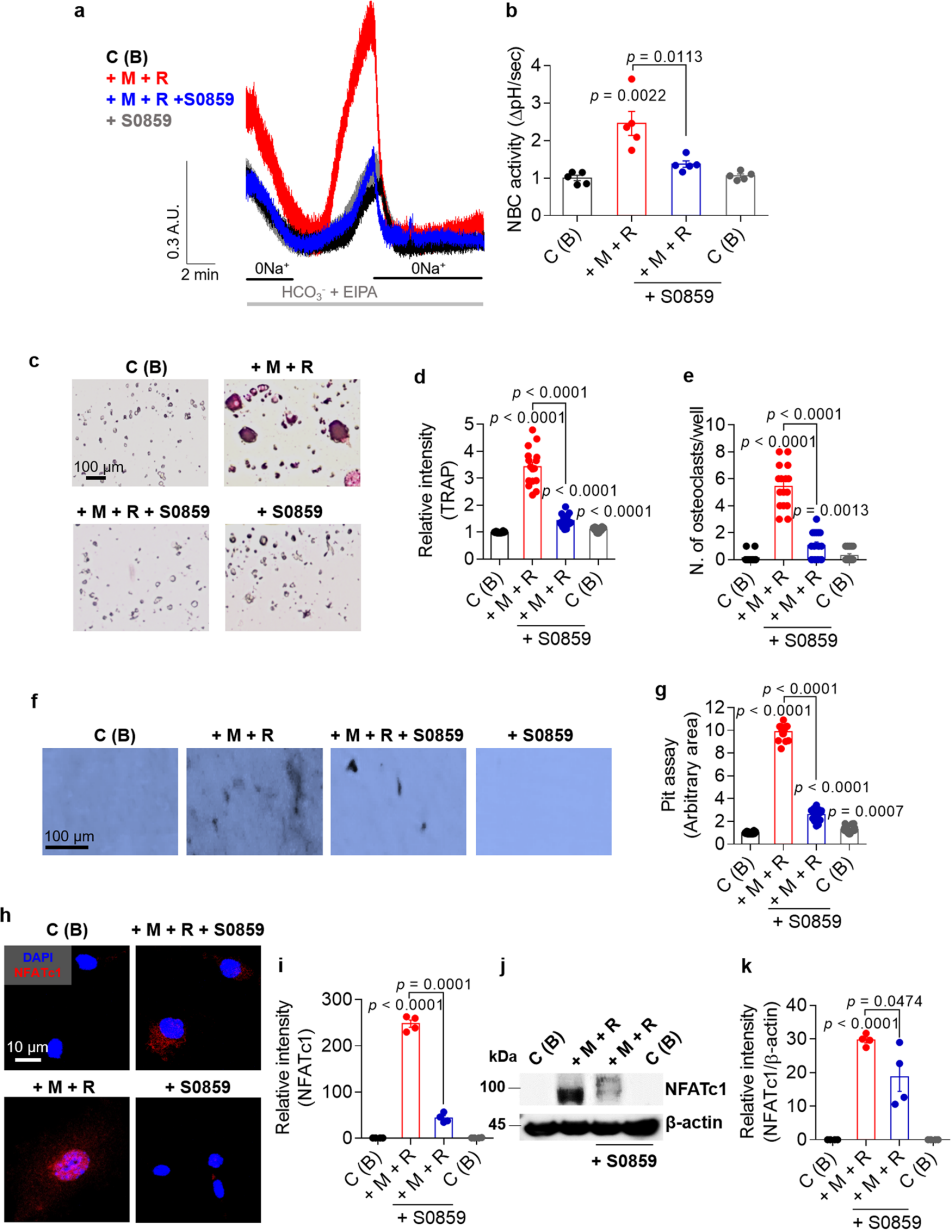
NBC inhibition attenuates osteoclastogenesis and bone resorption in BMMs. **a** NBC activity was assessed by measuring the changes in the pH_i_ values of M-CSF- and RANKL-stimulated BMMs treated with or without 20 μΜ S0859 for 24 h. **b** An analysis of NBC activity in BMMs. The bars represent the mean ± s.e.m. (*n* = 5). **c** Representative images of TRAP staining in osteoclasts derived from BMMs under the indicated conditions; M-CSF was administered for 3 days, followed by an additional 4 days of M-CSF treatment. The experiments were conducted in the presence or absence of 20 μΜ S0859 for 3 days. Scale bar, 100 μm. **d** An analysis of the relative TRAP intensity in osteoclasts derived from BMMs. The bars represent the mean ± s.e.m. (*n* = 16). **e** An analysis of the relative number of TRAP-positive osteoclasts derived from BMMs. The bars represent the mean ± s.e.m. (*n* = 16). **f** Bone resorption pit assay of BMMs cultured with dentin discs and subjected to the indicated conditions. BMMs were cultured with dentin discs and treated with M-CSF for 4 days, followed by exposure to 20 μM S0859 for 3 days. Scale bar, 100 μm. **g** An analysis of lacunae (dark areas) formed by BMMs. The bars represent the mean ± s.e.m. (*n* = 16). **h** Immunofluorescence staining for NFATc1 (red) and DAPI (blue) in BMMs subjected to the indicated stimuli. BMMs were first treated with M-CSF for 3 days, followed by an additional 4 days of treatment with M-CSF in the presence or absence of 20 μM S0859. Scale bar, 10 μm. **i**, An analysis of the normalized intensity (total intensity/measuring area) of NFATc1 in BMMs. The bars represent the mean ± s.e.m. (*n* = 4). **j** NFATc1 protein levels in BMMs subjected to the indicated conditions. The cells were first treated with M-CSF for 3 days, followed by an additional 4 days of treatment with M-CSF in the presence or absence of 20 μM S0859. β-Actin was used as a loading control. **k** An analysis of the band intensity of the NFATc1 protein in BMMs. The bars represent the mean ± s.e.m. (*n* = 4).

We determined the role of NBC in primary cultured BMMs treated with or without the NBC inhibitor S0859. Treatment with M-CSF and RANKL increased NBC activity; however, in the presence of S0859, M-CSF + RANKL-mediated NBC activity was reduced in BMMs (Fig. 7a, b). Treatment with 40 μM S0859 did not affect the viability of BMMs (Supplementary Fig. 4b). We determined whether the attenuated NBC activity in BMMs induces cellular fusion and bone resorption. Treatment with S0859 markedly attenuated M-CSF + RANKL-stimulated multinucleated cell fusion and decreased the number of mature osteoclasts (Fig. 7c–e) and bone resorption (Fig. 7f, g). However, treatment with S0859 did not affect the viability of FLSs (Supplementary Fig. 5). BMMs treated with or without S0859 were immunostained for NFATc1 to assess NFATc1 expression. M-CSF + RANKL-treated BMMs presented increased nuclear localization and increased protein expression of NFATc1; however, treatment with S0859 reduced NFATc1 expression (Fig. 7h–k). These results indicate that NBC inhibition attenuates osteoclast differentiation and bone resorption activity.

### NBC inhibitor S0859-exposed osteoclast medium does not induce FLS migration

**Fig. 8:**
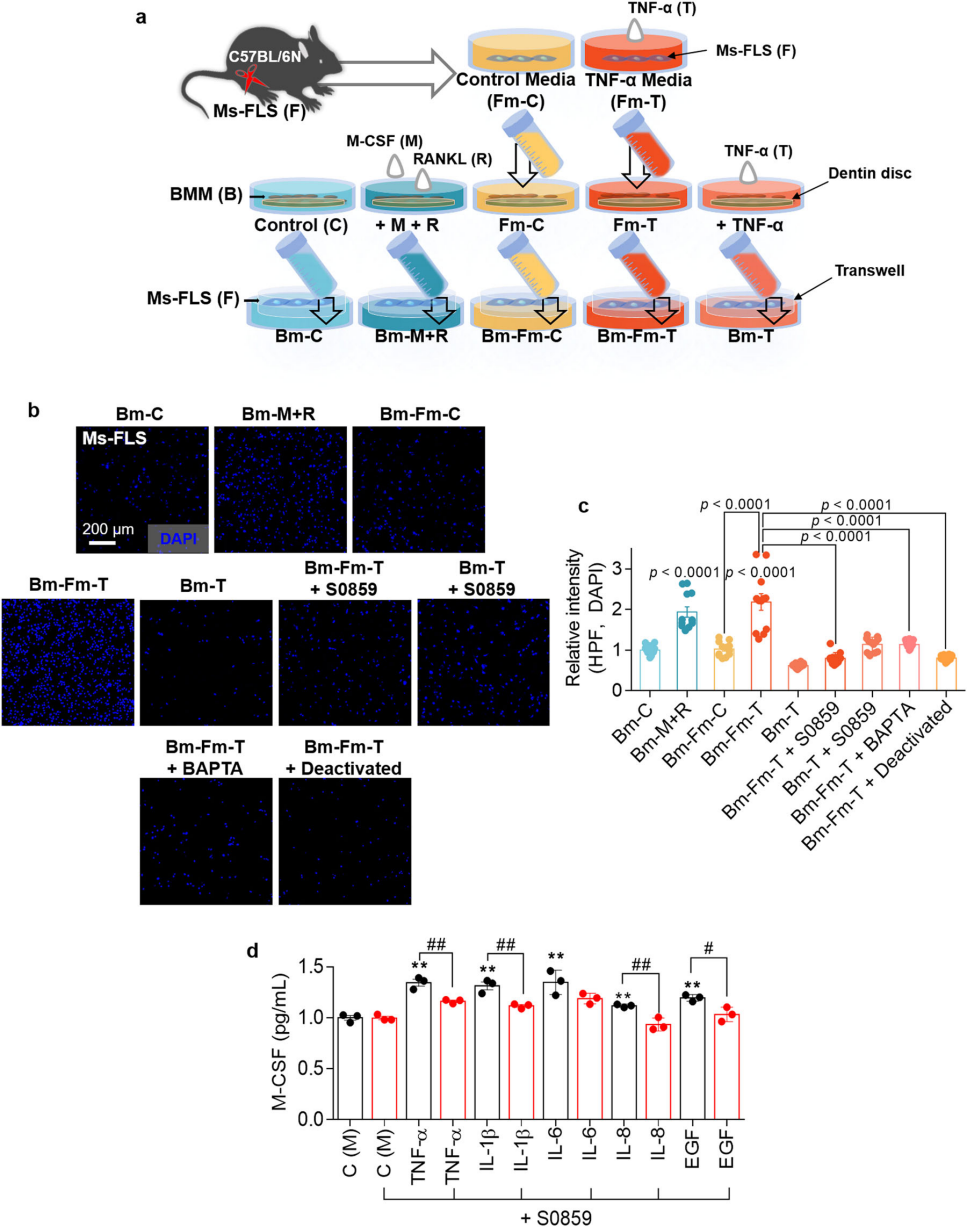
NBC inhibitor S0859-exposed osteoclast medium does not induce FLS migration. **a** The schematic procedure of primary Ms-FLS migration cultured in the presence of the BMM medium-mediated stimuli. **b** Migration assays of isolated Ms-FLSs subjected to the indicated conditions for 6 h. Fluorescence staining with DAPI (blue). Scale bar, 200 μm. **c** An analysis of the total DAPI intensity was performed to assess Ms-FLS migration under the indicated conditions. The bars represent the mean ± s.e.m. (*n* = 12). **d** The concentration of M-CSF secreted from Ms-FLSs subjected to the indicated conditions. The bars represent the mean ± s.e.m. (*n* = 3, *P < 0.05, **P < 0.01, ^#^*P* < 0.05, ^##^*P* < 0.01).

BMMs were exposed to FLS medium obtained under five different conditions and cultured for 8–9 days until they reached the mature state to verify the role of NBC in BMM fusion and bone resorption after treatment with or without S0859. New FLSs were exposed to dentin disc-containing BMM medium in the lower chamber of the transwell system to determine the effects of the factors released by mature osteoclasts on FLS migration. We illustrated the experimental approach in Fig. 8a. FLS migration was increased in response to mature BMM-conditioned medium, Bm-M + R and Bm-Fm-T; however, FLS migration, mediated by the dentin components released from mature BMMs into the medium, was inhibited in the presence of S0859 (Fig. 8b, c). As shown in Fig. 4f, h, Ca^2+^ chelation or deactivated medium Bm-Fm-T reduced FLS migration (Fig. 8b, c). Furthermore, we determined whether NBC inhibition affects the expression of the osteoclast differentiation factor M-CSF by stimulating primary cultures of Ms-FLSs with various cytokines. Treatment with S0859 attenuated the cytokine-mediated M-CSF increases (Fig. 8d). These results indicate that NBC inhibition has inhibitory effects on cytokine-mediated FLS migration and osteoclast maturation.

### Inhibitory effect of S0859 on bone resorption in the CIA mouse model

**Fig. 9:**
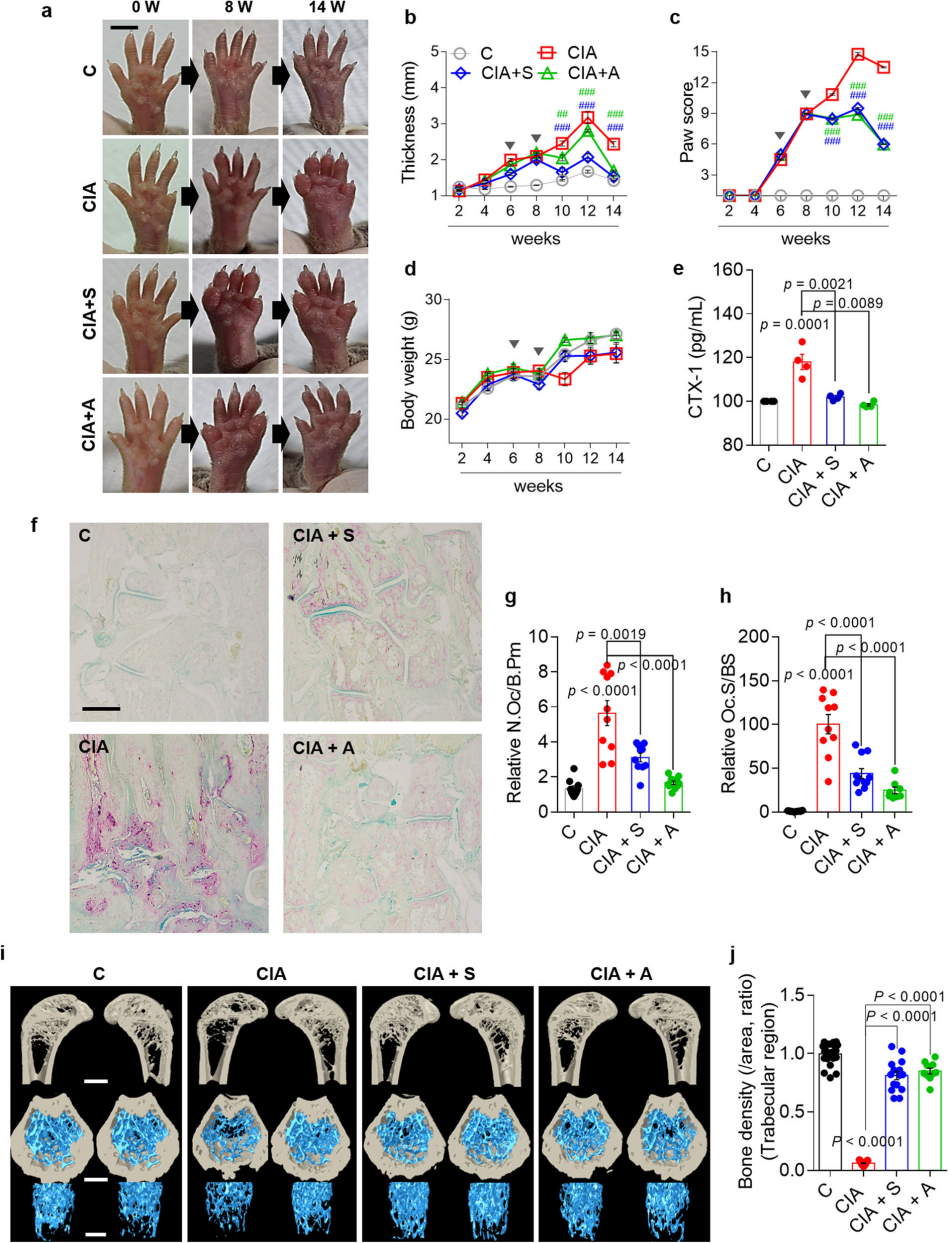
Inhibitory effect of S0859 on bone resorption in the CIA mouse model. **a** Representative images of the paws of mice treated with the indicated stimuli captured at 0, 8 and 14 weeks. Scale bar, 5 mm. **b** The paw thickness (mm) of mice in all groups was analyzed every 2 weeks. The bars represent the mean ± s.e.m. (^##^*P* < 0.01, ^###^*P* < 0.001). **c** Paw scores of all the groups were analyzed every 2 weeks. The bars represent the mean ± s.e.m. (^##^*P* < 0.01, ^###^*P* < 0.001). **d** Body weight was analyzed every 2 weeks in all groups. The bars represent the mean ± s.e.m. In **b**–**d** the arrowheads represent the drug administration points. **e** The serum concentration of secreted CTX-1 in mice subjected to the indicated treatments. The bars represent the mean ± s.e.m. **f** Representative images of TRAP-stained plantar bones obtained from mice subjected to the indicated treatments after 14 weeks. Scale bar, 500 μm. **g**, **h** An analysis of the number of osteoclasts (N.Oc)/bone perimeter (B.Pm) (**g**) and the osteoclast surface (Oc.S)/bone surface (BS) (**h**) in the indicated groups. The bars represent the mean ± s.e.m. compared with the control group. **i** Representative micro-CT images of trabecular bone in the femur of mice from the indicated groups. The top images show vertical sections, whereas the bottom images show horizontal sections. Scale bars, 500 μm. **j** An analysis of the bone density of the trabecular region of the femur from mice in the indicated groups. The bars represent the mean ± s.e.m. compared with the control group. C, control; S, S0859; A, adalimumab.

We determined whether the inhibition of NBC disrupted the infinite cytokine feedback loop and bone damage in a mouse model of CIA. The process used to establish the CIA mouse model is shown in Supplementary Fig. 6. The CIA model mice presented increased paw thickness and paw scores, with gradually increasing body weights observed 4 weeks after arthritis development (Fig. 9a–d and Supplementary Fig. 7a–d). Paw swelling was observed in the CIA group; however, this swelling was reduced at 14 weeks in the S0859-injected or adalimumab-injected CIA groups (Fig. 9a–c). Adalimumab, which is a monoclonal TNF antibody widely used to treat RA^24,25^, was used as a positive control. The serum levels of C-terminal telopeptide of type I collagen (CTX-1), a biomarker of bone resorption^26^, were measured to verify bone resorption in the mouse model. Elevated CTX-1 levels were detected in the CIA group, indicating increased bone resorption, whereas CTX-1 levels were significantly reduced in the S0859-injected or adalimumab-injected CIA groups (Fig. 9e). In addition, bone slice samples were stained with TRAP to verify whether NBC inhibition modulates osteoclastogenesis in mouse bones. The CIA group presented increased numbers of osteoclast nuclei and osteoclastogenic areas, whereas the S0859-injected or adalimumab-injected CIA groups presented reduced osteoclastogenic areas (Fig. 9f–h and Supplementary Fig. 7e–g). Moreover, bone porosity was measured using micro-CT to verify the inhibitory effect of S0859 on bone remodeling in the CIA mouse model. The bone density of the CIA group was reduced, whereas that of the S0859-injected or adalimumab-injected CIA group recovered to control levels (Fig. 9i, j and Supplementary Fig. 7h, i). The effects of S0859 alone on the paw thickness, paw score, body weight, TRAP staining of bone slices, and micro-CT images in control mice are shown in Supplementary Fig. 7. Treatment of control mice with S0859 resulted in outcomes similar to untreated control mice (Supplementary Fig. 7). These results indicate that NBC inhibition reduces swelling, pathological changes associated with RA, and bone-absorbing osteoclastogenic function.

## Discussion

**Fig. 10:**
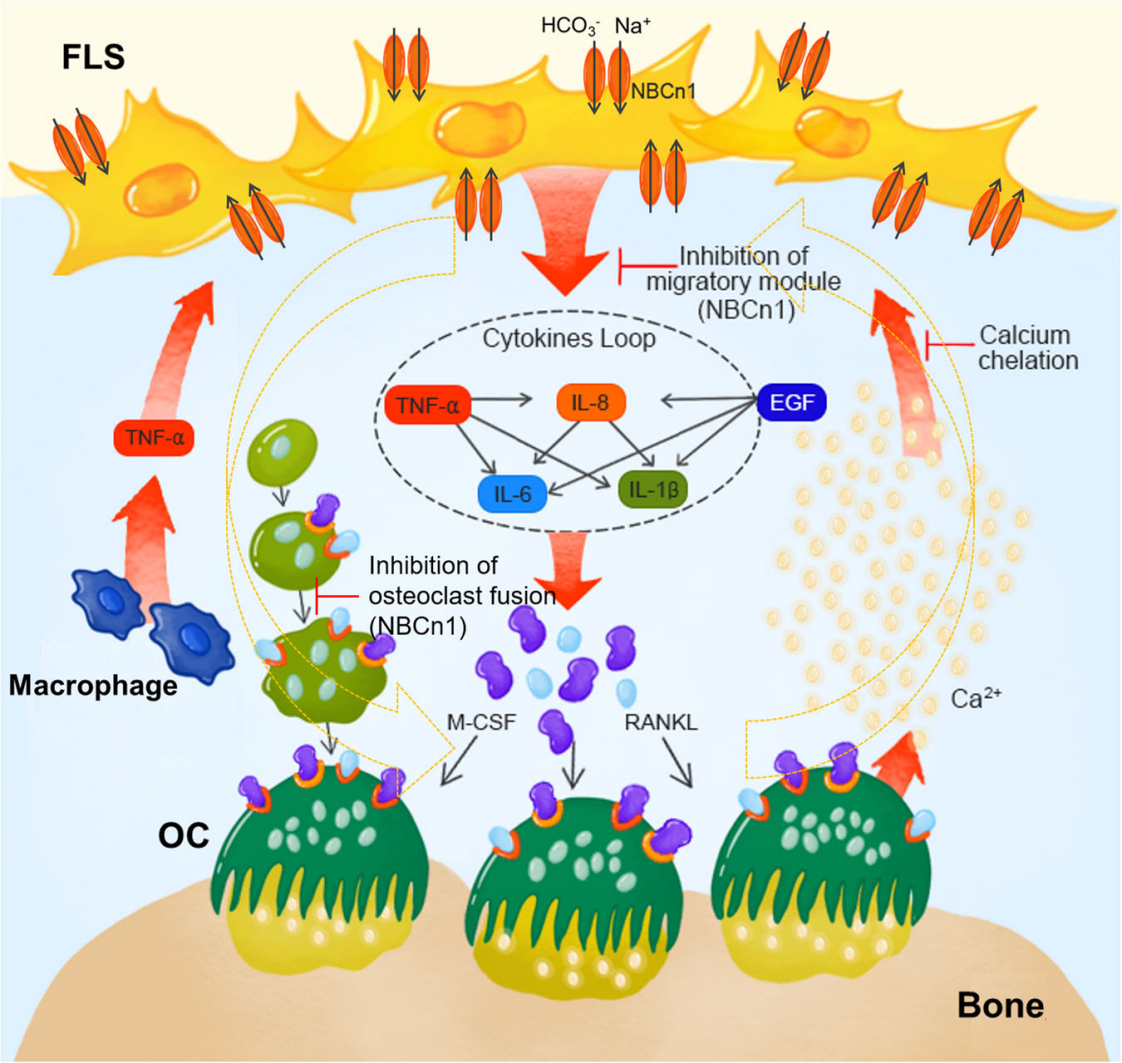
Schematic illustration of the cytokine feedback loop-mediated crosstalk between FLSs and osteoclasts. The released inflammatory cytokines and Ca^2+^, which are main components in inflammatory synovium enhanced the bone resorption factor M-CSF from RA-FLSs. M-CSF derived from RA-FLSs promoted osteoclast-induced bone resorption, which increased the release of Ca^2+^ from the bone. Increased Ca^2+^ and cytokine levels simultaneously increased FLS activation, suggesting that a cytokine feedback loop is continuously generated, which subsequently exacerbates RA. The open-dotted curved arrows represent the cytokine feedback loop between FLSs and osteoclasts through the involvement of releasing components such as cytokines and Ca^2+^. OC, osteoclast.

In this study, we investigated the Ca^2+^ and cytokine feedback loop between inflammation and bone resorption in RA-associated cellular and mouse models. Inflammatory cytokines and Ca^2+^ released from the bone increased the level of the bone resorption factor M-CSF in RA-FLSs. Factors released from RA-FLSs mediate the increased bone resorption activity of osteoclasts. The outputs of bone resorption subsequently induce FLS migration through the modulation of NBCn1. Secreted factors related to RA, such as inflammatory cytokines and Ca^2+^, mediated osteoclastogenesis, which was attenuated by inhibition of NBC activity. Furthermore, we verified the inhibitory effect of S0859 on bone damage and osteoclastogenic activity in the bones of a CIA mouse model. Previously, we reported that the aggressiveness of RA-FLSs is mediated by the recruitment of NBCn1 to the plasma membrane^12^. NBCn1, a bicarbonate transporter, provides a mechanical driving module for migration toward inflammatory signals^12^ and metastatic modulation in cancers^27^. Within the scope of inflammation, the inhibition of NBCn1 via treatment with S0859 improves RA severity in a CIA mouse model without changes in blood count, hepatic and renal toxicities^12^. In the present study, NBC inhibition by S0859 revealed dual inhibitory effects on the cytokine feedback loop and osteoclast differentiation. Our results also provide experimental insights into the direct interaction between FLSs and osteoclasts through a Ca^2+^ and cytokine feedback loop. A schematic illustration of the Ca^2+^ and cytokine feedback loop in both FLSs and osteoclasts is shown in Fig. 10.

Inflammatory cytokines and acidic stimuli increase ion transporter activity. Previously, we reported that NBCn1 is involved in the aggressiveness of FLSs^12^. Interestingly, inflammatory cytokine production and FLS migration are stimulated by increased Ca^2+^ levels, suggesting that the crosstalk between FLSs and osteoclasts is mediated by these factors. The factors released from the dentin disc, which mainly include Ca^2+^, are involved in FLS aggressiveness through the increased activity of the migratory module NBC. These FLSs and osteoclasts possess the same migratory apparatus, NBCn1. Inhibition of NBCn1 attenuates osteoclastogenesis, decreases the bone resorption area and inhibits the invasive phenotype of FLSs. Therefore, the modulation of NBCn1 in osteoclasts might be a new strategy to regulate bone homeostasis.

RA is a complex chronic inflammatory disease. Studies are ongoing to develop an optimized therapeutic target, recover individual variability and systemic comorbidities, reduce incomplete responses to medication and pathological complications, minimize side effects and reduce treatment costs related to RA^28,29^. The treatment strategies for RA involve various targets, including the inhibition of inflammatory cytokines by agents such as biological DMARDs^28^. In addition, synthetic DMARDs have been proposed to treat RA. For instance, Janus kinase inhibitors, such as upadacitinib, tofacitinib and baricitinib, offer advantages for patients who are nonresponsive to biologic DMARDs in RA treatment; however, some drugs have increased long-term safety concerns, such as cancer and cardiovascular risks, and comparisons of these drugs are still ongoing^30–32^. Nonsteroidal anti-inflammatory drugs or corticosteroids are also commonly used to treat pain and inflammation; however, their long-term application should be carefully considered due to cardiovascular issues^33,34^. In addition, therapies targeting TNF, a predominant cytokine, have been successfully developed over the past two decades^25^. However, patients who have contraindications or fail to respond to TNF therapy are recommended to receive other types of DMARD. Combination therapies using biologic or synthetic DMARDs such as methotrexate are also recommended^35–37^. Recently, combination approaches involving both the proteasome inhibitor delanzomib and the TNF inhibitor adalimumab have been proposed to treat RA^25^. Although DMARDs and their combination have been developed, the number of patients whose RA is difficult to treat is still as high as 20% (ref. ^38^). Thus, research on appropriate RA therapies and new targets are still ongoing, as identifying a complete cure remains a challenge. From this perspective, our study highlights the rationale for the need for an appropriate treatment for RA as an alternative to TNF therapy, such as Adalimumab therapy.

In this study, we determined that both inflammatory cytokines and Ca^2+^ release must be controlled to prevent the activation of FLSs, which are involved in RA pathology. In addition to Ca^2+^, FLS migration-associated components of the synovial fluid, such as dickkopf-1 (ref. ^39^), gastrin-releasing peptide^11^, class 3 semaphorins^40^ and anti-cyclic citrullinated peptide antibody^41^, have been detected in the RA synovium. Accordingly, the roles of these RA-related components in the Ca^2+^ and cytokine feedback loop should be characterized to develop new therapies for RA in the near future. In addition to the suppression strategy for each inflammatory factor in patients with RA, modulation of the crosstalk between FLSs and osteoclasts should be considered a pioneering strategy to attenuate RA severity and bone erosion and develop an appropriate drug treatment strategy.

## Animal studies

All experimental animal procedures were performed in accordance with the Gachon University guidelines and were approved by the Animal Care and Use Committee of Gachon University (LCDI-2022-0118).

## Supplementary information

Supplementary Information
