# Supplementary material for: Rheumatoid arthritis severity is mediated by crosstalk between synoviocytes and mature osteoclasts through a calcium and cytokine feedback loop
Source: Exp Mol Med. 2025 Feb 3;57(2):402–19. doi: 10.1038/s12276-025-01401-8 (PMC11873226; doi:10.1038/s12276-025-01401-8)
Supplement: Supplementary file 1 — Supplementary Information [file 12276_2025_1401_MOESM1_ESM.pdf]

Supplementary Figures

Rheumatoid arthritis severity is mediated by crosstalk between synoviocytes and mature osteoclasts through a calcium and cytokine feedback loop

Lee Eun Sun, Hyeong Jae Kim, Dongun Lee, Jung Yun Kang, Dong Min Shin*, and Jeong Hee Hong*

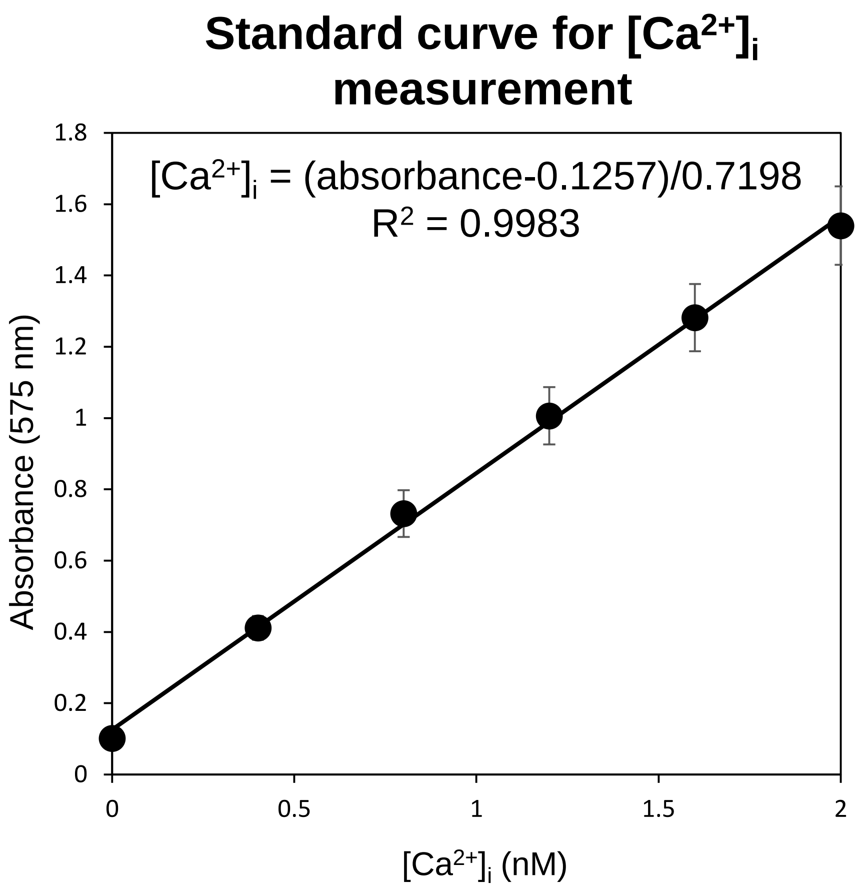

Supplementary Fig. 1. Standard curve for Ca^{2+} concentration

The standard curve used to determine Ca^{2+} concentration was obtained via a Ca^{2+} colorimetric assay. The following equation was used: $[Ca^{2+}]_i$ = (absorbance-0.1257)/0.7198.

Gene	KEGG
CSF1	has:1435
FYN	hsa:2534
IFNAR2	hsa:3455
IFNGR2	hsa:3460
IL1A	hsa:3552
IL1B	hsa:3553
LCP2	hsa:3937
NFKB1	hsa:4790
NFKB2	hsa:4791
NFKBIA	hsa:4792
PIK3CB	hsa:5291
PPP3CC	hsa:5533
RELB	hsa:5971
TRAF2	hsa:7186
TYK2	hsa:7297

Abbreviations: **CSF1**: macrophage colony-stimulating factor 1; **FYN**: src family tyrosine kinase; **IFNAR2**: interferon alpha and beta receptor subunit 2; **IFNGR2**: interferon gamma receptor 2; **IL1A**: interleukin 1 alpha; **IL1B**: interleukin 1 beta; **LCP2**: lymphocyte cytosolic protein 2; **NFKB1**: nuclear factor kappa B subunit 1; **NFKB2**: nuclear factor kappa B subunit 2; **NFKB1A**: nuclear factor kappa B inhibitor alpha; **PIK3CB**: phosphatidylinositol-4,5,-bisphosphate 3-kinase catalytic subunit beta; **PPP3CC**: protein phosphatase 3 catalytic subunit gamma; **RELB**: NF-KB subunit; **TRAF2**: tumor necrosis factor receptor associated factor 2; **TYK2**: tyrosine kinase 2

Supplementary Fig. 2. Osteoclast differentiation-related gene list

The table lists the KEGG analysis results of the upregulated genes related to osteoclast differentiation.

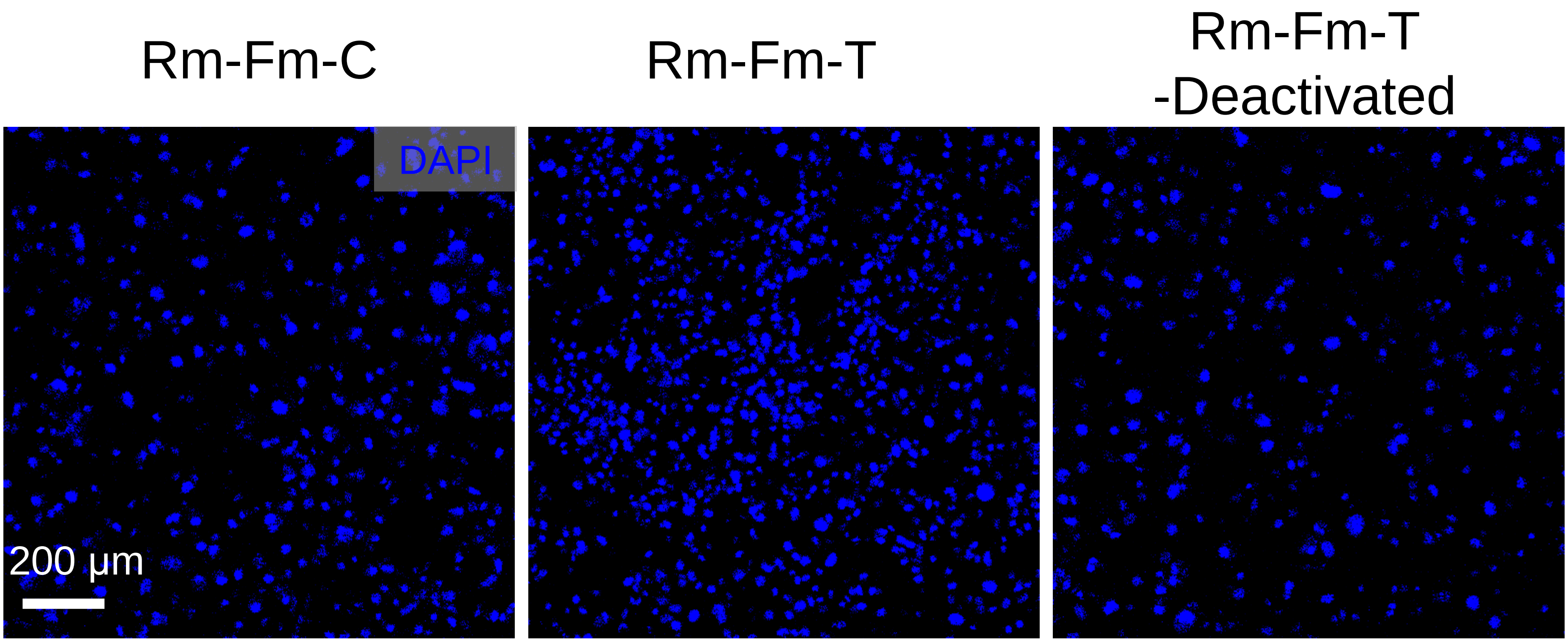

Supplementary Fig. 3. Deactivated TNF-α-treated FLS media did not induce FLS migration

Migration assays of mouse FLS isolated after 6 h under the indicated conditions are shown in Fig 4F. Fluorescence staining with DAPI (blue). Scale bar represents 200 μm.

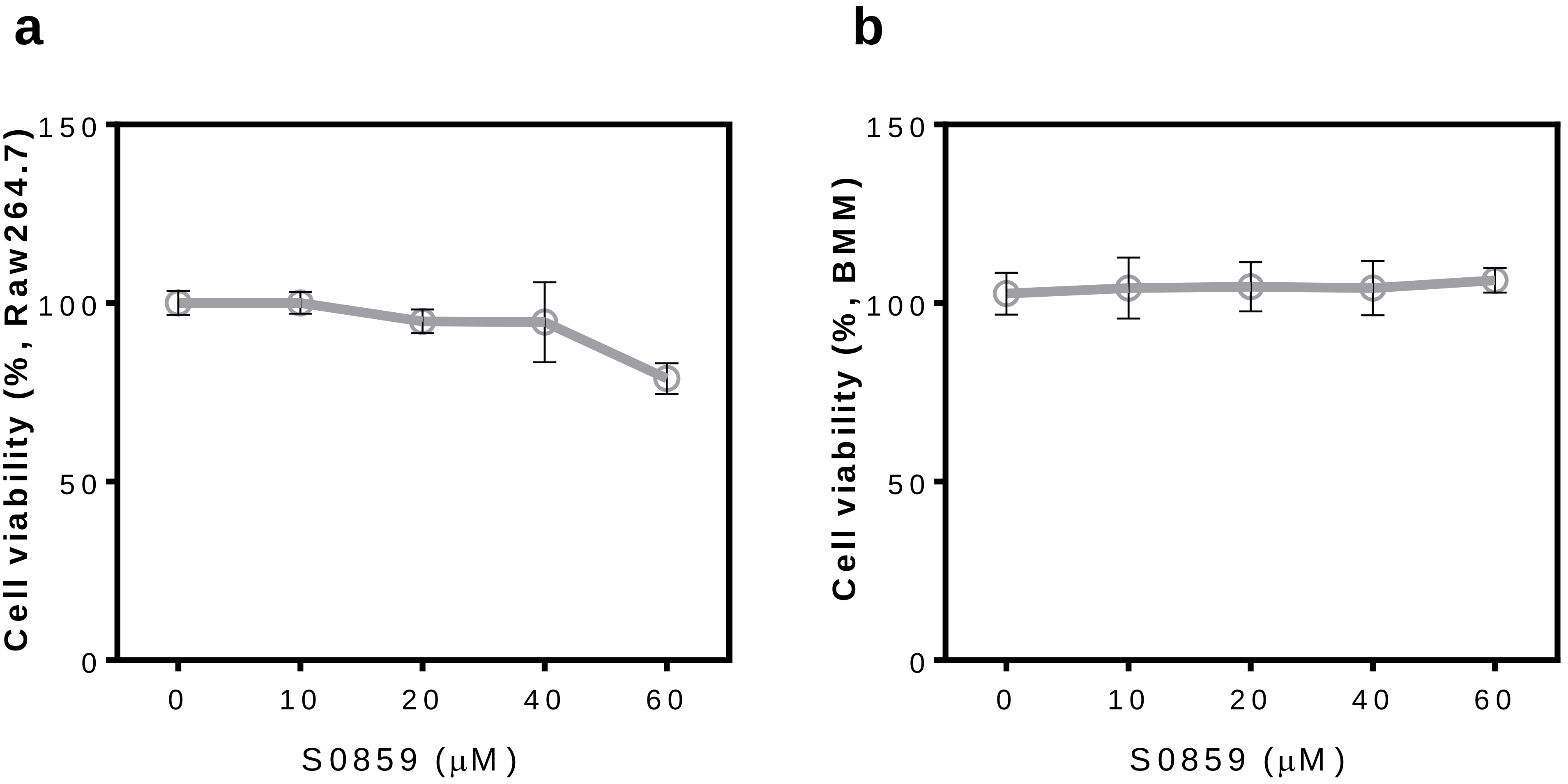

Supplementary Fig. 4. Cellular stability of S0859 for OC cells

(a, b) Cell viability assay of Raw264.7 (a) and BMM (b) in the presence of different doses of S0859 (0, 10, 20, 40, and 60 μM) for 24 h.

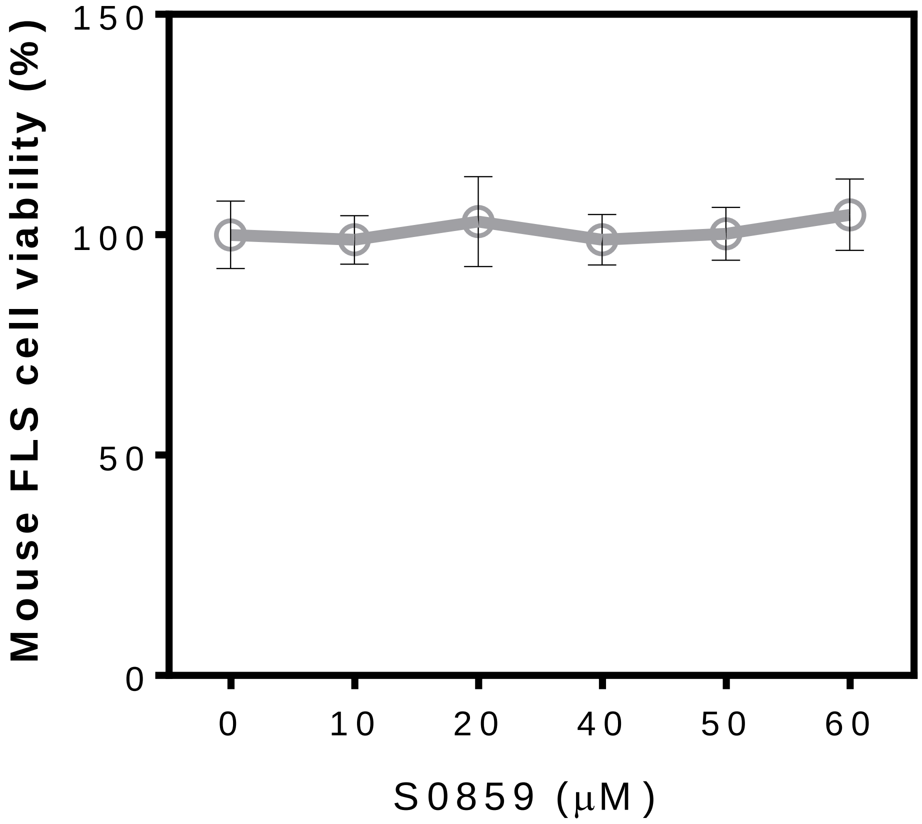

Supplementary Fig. 5. Cellular stability of S0859 for Ms-FLS

Cell viability assay of Ms-FLS in the presence of different doses of S0859 (0, 10, 20, 40, and 60 μM) for 24 h.

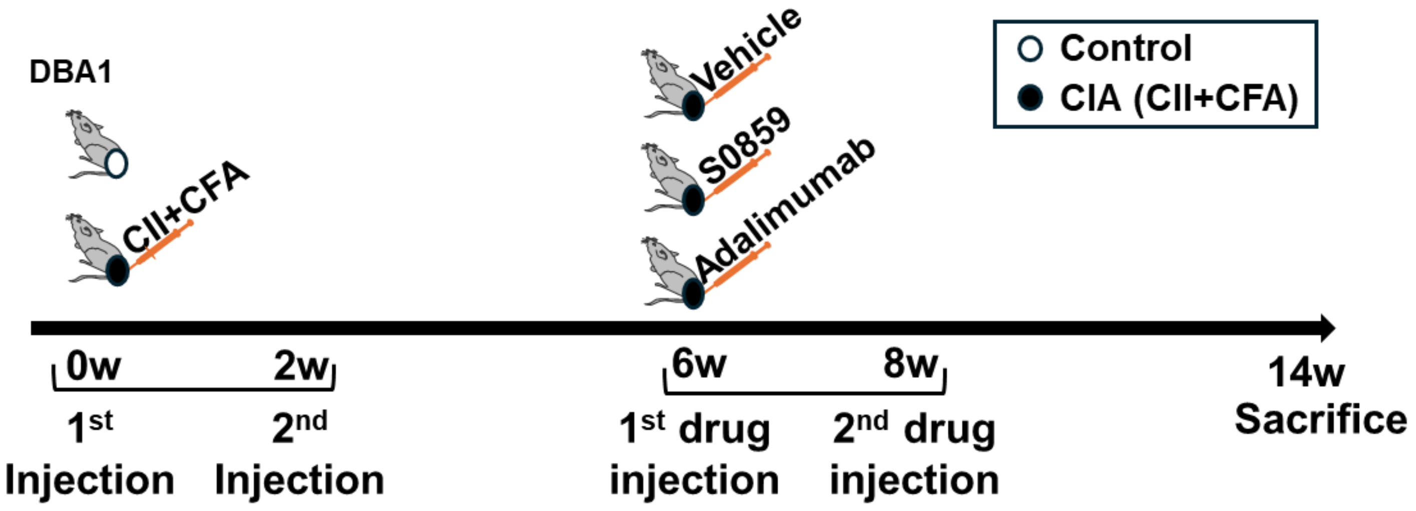

Supplementary Fig. 6. Schematic representation of the experimental timeline for the induction and treatment of CIA in DBA/1 mice

The timeline illustrates the protocol for inducing Collagen-Induced Arthritis (CIA) in DBA/1 mice with CII + CFA injections at 0 week and 2 weeks. Following arthritis induction, vehicle, S0859, and Adalimumab were administered at 6 weeks and 8 weeks (first and second drug injections, respectively). The mice were sacrificed at 14 weeks for analysis. The diagram was distinguished between control (○) and CIA (CII+CFA) (●) groups.

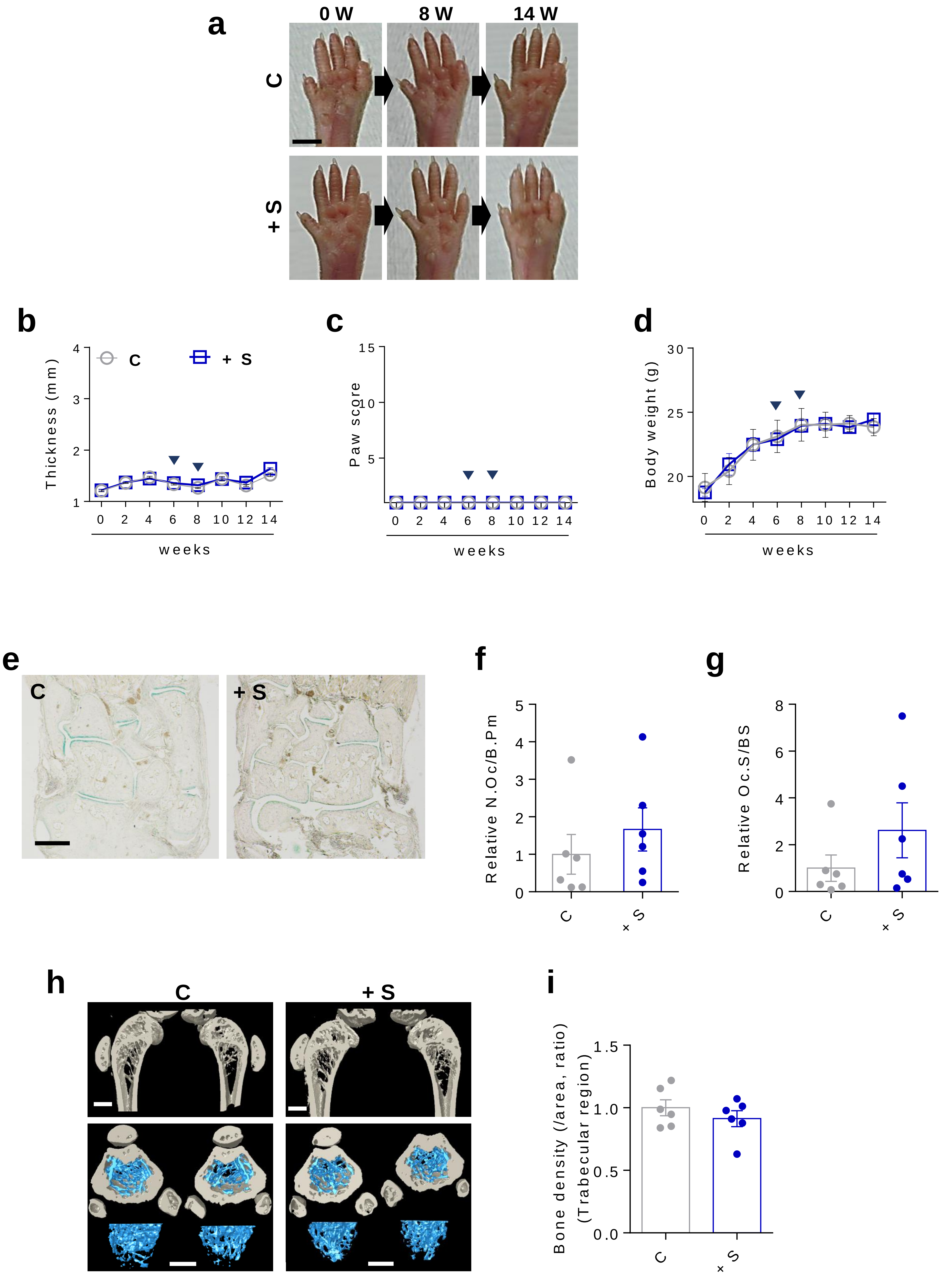

a
0 W
8 W
14 W
C
+ S
b
Thickness (mm)
C
+ S
weeks
c
Paw score
weeks
d
Body weight (g)
weeks
e
C
+ S
f
Relative N.Oc/B.Pm
C
+ S
g
Relative Oc.S/BS
C
+ S
h
C
+ S
i
Bone density (/area, ratio)
(Trabecular region)
C
+ S

Supplementary Fig. 7. Treatment of S0859 in bone resorption in the control mouse

(a) Representative images of control mouse paws with or without S0859 at 0, 8, and 14 weeks. The scale bar represents 5 mm. **(b)** Analysis of the paw thickness (mm) every 2 weeks in all groups. Bars represent mean ± SEM. **(c)** Analysis of the paw score every 2 weeks in all groups. Bars represent mean ± SEM. **(d)** Analysis of the body weight every 2 weeks in all groups. Bars represent mean ± SEM. Arrow heads represent drug administered points (B-D). **(e)** Representative images of TRAP-stained plantar bones subjected to the indicated conditions after 14 weeks. Scale bar represents 200 μm. **(f, g)** Analysis of the number of osteoclast (N. Oc)/bone perimeter (B.Pm) (f) and the osteoclast surface (Oc. S)/bone surface (BS) (g) under the indicated conditions. Bars represent mean ± SEM relative to the control. **(h)** Representative micro-CT images of mouse femur trabecular bone under the indicated conditions. The upper images show the vertical section while the bottom images show the horizontal section. Scale bars represent 500 μm. **(i)** Analysis of the bone density of mouse femur trabecular region under the indicated conditions. Bars represent mean ± SEM relative to the control. Abbreviations. Control (C), S0859 (S).
